# A Molecular-Level Account of the Antigenic Hantaviral Surface

**DOI:** 10.1016/j.celrep.2016.03.082

**Published:** 2016-04-21

**Authors:** Sai Li, Ilona Rissanen, Antra Zeltina, Jussi Hepojoki, Jayna Raghwani, Karl Harlos, Oliver G. Pybus, Juha T. Huiskonen, Thomas A. Bowden

**Affiliations:** 1 Division of Structural Biology, Wellcome Trust Centre for Human Genetics, University of Oxford, Roosevelt Drive, Oxford OX3 7BN, UK; 2 Department of Virology, Haartman Institute, University of Helsinki, 00014 Helsinki, Finland; 3 Department of Zoology, University of Oxford, South Parks Road, Oxford OX1 3PS, UK

## Abstract

Hantaviruses, a geographically diverse group of zoonotic pathogens, initiate cell infection through the concerted action of Gn and Gc viral surface glycoproteins. Here, we describe the high-resolution crystal structure of the antigenic ectodomain of Gn from Puumala hantavirus (PUUV), a causative agent of hemorrhagic fever with renal syndrome. Fitting of PUUV Gn into an electron cryomicroscopy reconstruction of intact Gn-Gc spike complexes from the closely related but non-pathogenic Tula hantavirus localized Gn tetramers to the membrane-distal surface of the virion. The accuracy of the fitting was corroborated by epitope mapping and genetic analysis of available PUUV sequences. Interestingly, Gn exhibits greater non-synonymous sequence diversity than the less accessible Gc, supporting a role of the host humoral immune response in exerting selective pressure on the virus surface. The fold of PUUV Gn is likely to be widely conserved across hantaviruses.

## Highlights

• We describe the high-resolution crystal structure of a hantaviral Gn ectodomain
• Electron cryotomography analysis reveals the ultrastructure of Gn-Gc assembly
• X-ray fitting and mapping analysis reveals the antigenic hantavirus surface
• The Gn fold is likely to be widely conserved across this group of viruses

## Graphical Abstract

**Figure:**
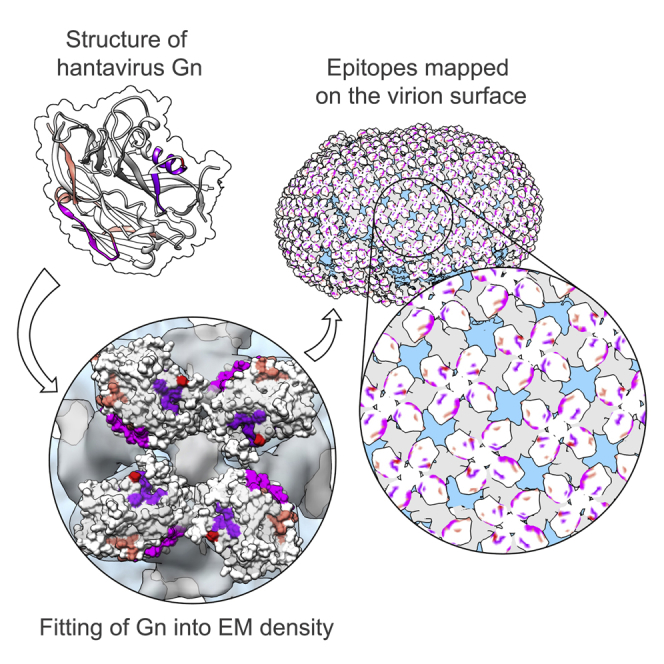

## Teaser

Li et al. combine X-ray crystallography with electron cryotomography and sub-tomogram averaging to determine the organization of hantaviral glycoproteins on the mature virion envelope. This work provides a molecular-level description of the antigenic surface for a group of widespread and deadly viral pathogens.

## Introduction

Hantaviruses, from the family *Bunyaviridae*, constitute a genus of human pathogens with a near-worldwide distribution (Jonsson et al., 2010). These viruses chronically and asymptomatically infect rodents, shrews, moles, and bats. Cross-species transmission to humans, primarily via aerosolized animal excreta, can lead to severe diseases (Jonsson et al., 2010, Lee and Johnson, 1982, Nuzum et al., 1988). Clinical symptoms of hantavirus infection usually manifest two to three weeks following initial exposure and lead to either hantavirus pulmonary syndrome (HPS) or hemorrhagic fever with renal syndrome (HFRS) (Lednicky, 2003). The case-mortality rates typically range from 0.1 to 10 % for HFRS to up to 40% for HPS (Vaheri et al., 2013).

Hantaviruses have a lipid-bilayer envelope, and their negative-sense RNA genome is divided into S, M, and L segments. The ∼1,150-amino-acid glycoprotein precursor is encoded by the M segment (Schmaljohn et al., 1987) and is co-translationally cleaved by the cellular signal peptidase complex at the conserved “WAASA” sequence (Löber et al., 2001) into two structural glycoprotein components, Gn (∼70 kDa) and Gc (∼55 kDa). Low resolution three-dimensional (3D) structures of Tula (TULV) and Hantaan virus spike complexes, derived by electron cryomicroscopy studies and combined with biochemical analysis, revealed that Gn and Gc form square-shaped oligomeric complexes on the virion envelope (Battisti et al., 2011, Hepojoki et al., 2010, Huiskonen et al., 2010).

Similar to the Gc from Rift Valley fever virus (genus *Phlebovirus*), another *Bunyaviridae* family member (Dessau and Modis, 2013), the hantaviral Gc is expected to form a class-II membrane fusion protein fold (Tischler et al., 2005). The fold of the Gn ectodomain, on the other hand, is unknown. Following an initial interaction between a cell-surface receptor and the hantaviral Gn-Gc complex, the virus is endocytosed and fusion of the cellular and viral membranes is thought to occur via a pH-dependent process (Acuña et al., 2015, Jin et al., 2002). Several cell-surface glycoproteins, including integrins, the decay-accelerating factor (DAF/CD55), and complement receptor gC1qR, have been suggested as viral entry receptors (Buranda et al., 2010, Choi et al., 2008, Gavrilovskaya et al., 1998, Raymond et al., 2005).

We determined the crystal structure of the Gn ectodomain from Puumala virus (PUUV), a hantavirus endemic in common vole populations throughout Eurasia and responsible for nephropathia epidemica, a mild form of HFRS. Using electron cryotomography (cryo-ET), we resolved the structure of the envelope glycoprotein spike complex from the closely related apathogenic Tula virus (TULV) to 16 Å resolution. This facilitated fitting of the Gn to the four membrane-distal lobes of the spike, a placement corroborated by estimation of synonymous and non-synonymous nucleotide substitutions in PUUV sequences and mapping of previous biochemical analyses on the structure. Combined with antibody epitope mapping, these data provide a detailed description of the antigenic hantaviral surface.

## Results

### Expression of the PUUV Gn ectodomain

Similar to other hantaviruses (Schmaljohn et al., 1987), PUUV Gn encodes a signal sequence (residues 1−24) (Petersen et al., 2011), an N-terminal ectodomain (residues 25−504), a predicted transmembrane region (residues 505−526) (Krogh et al., 2001), and a C-terminal cytoplasmic domain (residues 527−658). To facilitate soluble protein expression, a PUUV Gn construct (residues 29−383) was truncated by ∼120 residues prior to the C-terminal transmembrane helix and transiently expressed in HEK293S cells. As observed by size-exclusion chromatography in both neutral (pH 8.0) and acidic (pH 5.0) conditions (Figure S1), PUUV Gn is a monomer in solution, consistent with the hypothesis that residues 450 onward contribute to tetramer formation (Hepojoki et al., 2010).

### Structure of PUUV Gn

**Figure 1:**
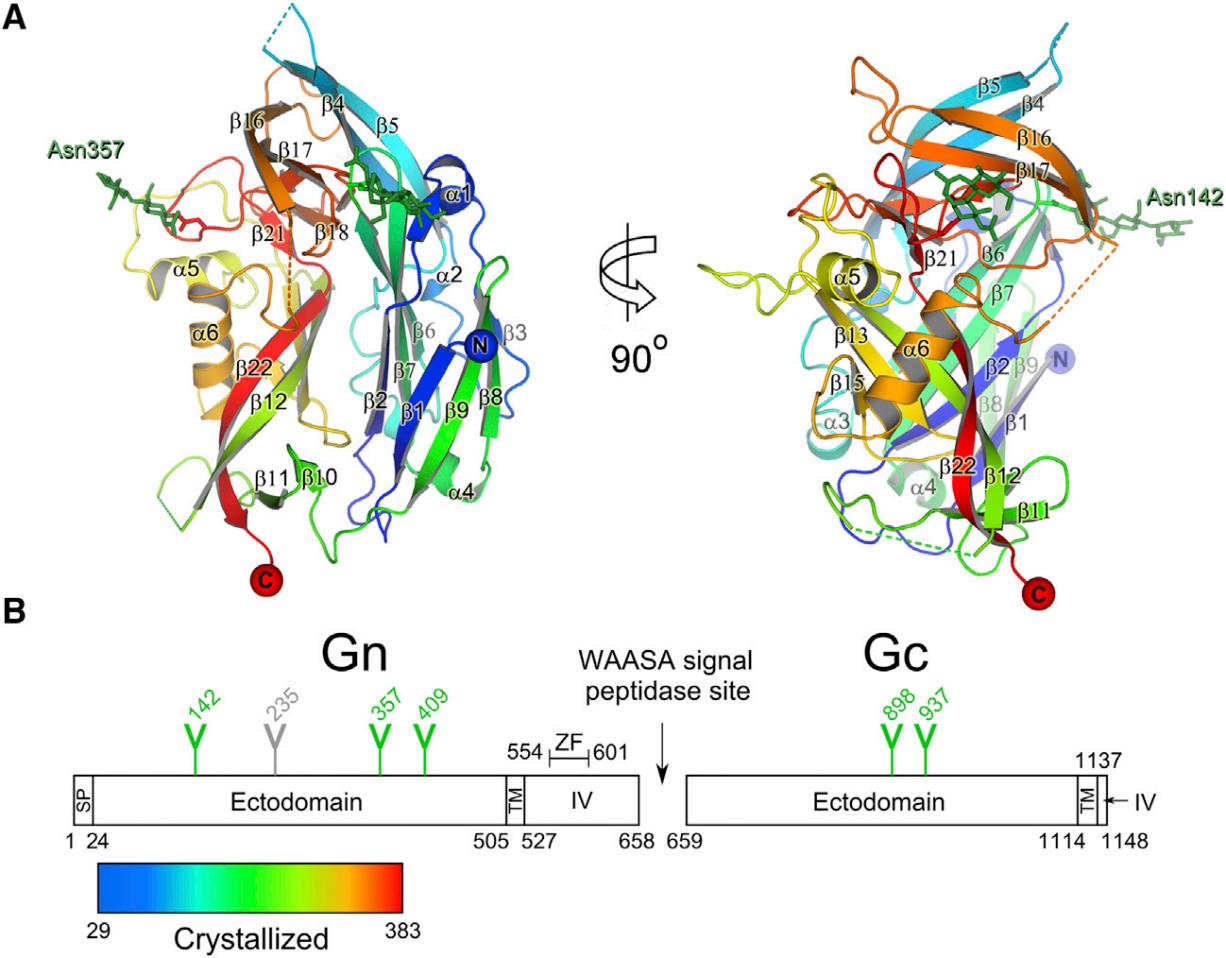
Crystal Structure of the Puumala Gn Ectodomain (A) A ribbon representation of Puumala (PUUV) Gn colored from blue (N terminus) to red (C terminus). N-linked glycans are shown as green sticks. (B) Domain schematic of PUUV glycoprotein precursor with the signal peptide (SP), ectodomain, transmembrane domain (TM), intravirion domain (IV), zinc finger (ZF), and WAASA signal peptidase cleavage site shown (produced with DOG; Ren et al., 2009). Y-shaped symbols designate N-linked glycosylation sites. The location of the additional putative N-linked glycosylation site at Asn235 in Hantaan virus (Lys243 in PUUV) is indicated in gray. See also Figures S1 and S2.

The crystal structure of PUUV Gn was determined to 2.3 Å resolution using the single-wavelength anomalous diffraction (SAD) method (Table 1). PUUV Gn forms an α/β fold (∼40 kDa), consisting of five α helices, a 3_10_ helix, and twenty-two β strands. The β strands assemble to form five β sheets, which associate together by the formation of a β sandwich (Figure 1). The two molecules of PUUV Gn present in the crystal asymmetric unit are almost identical, with differences being limited to solvent-accessible loops (0.7 Å root mean square deviation in equivalent Cα positions over 327 residues; Figure S1). For both molecules in the asymmetric unit, three loops (residues 92−102, 204−208, and 292−300) were not clearly visible in the electron density, and it is likely that these residues are either naturally flexible or require an associated protein, such as neighboring Gn/Gc protomers, to impose order. No higher order oligomerization was detected from the crystallographic packing, supporting the hypothesis that the Gc glycoprotein and/or C-terminal regions of the Gn may, in part, be required for tetramer formation (Hepojoki et al., 2010). The PUUV Gn fold is stabilized by seven intra-domain disulfide bonds, a pattern well-conserved among hantaviruses (Figure S2). This, together with the comparatively high level of sequence conservation across rodent-borne hantaviruses (>50%; Figure S3), suggests that the observed fold is a defining feature of the genus.

The presence of N-linked, predominantly high-mannose glycosylation on the hantaviral Gn is another shared feature across the genus (Figure S2) (Johansson et al., 2004, Shi and Elliott, 2004). The PUUV Gn sequence exhibits N-linked glycosylation sequons at Asn142, Asn357, and Asn409 (which was not included in the crystallized construct). Electron density was observed at both Asn142 and Asn357 (Figure S1), with the glycans extending away from the protein surface. It is likely that the well-ordered nature of these moieties is induced by stabilizing contacts with adjacent molecules in the crystal. These data suggest that both N-linked glycan sites are occupied on PUUV virions.

### Structure of the Hantaviral Surface

Apathogenic TULV is one of the closest known relatives to PUUV and a model for hantavirus ultrastructure (Huiskonen et al., 2010). We set out to study the architecture of Gn/Gc glycoprotein complexes to facilitate localization of our Gn crystal structure on the virion. Combining established techniques in cryo-ET and sub-tomogram averaging (Huiskonen et al., 2014) with direct-detector technology (Bammes et al., 2012), we improved the resolution of the TULV Gn/Gc spike structure from 36 Å (Huiskonen et al., 2010) to 16 Å (Table 2).

**Figure 2:**
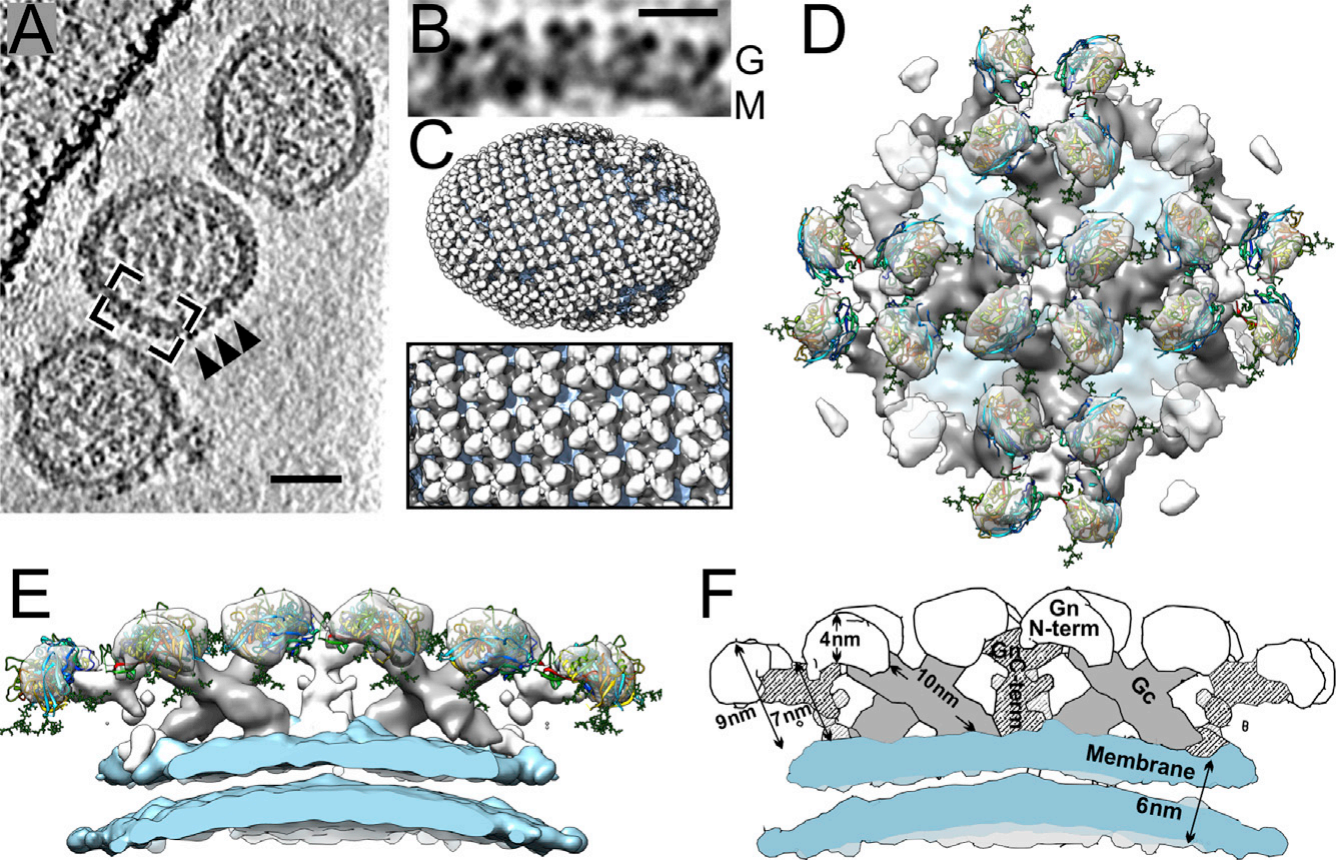
Organization of Tula Virion (A) Low-pass filtered computational section from a tomographic reconstruction of three Tula virus (TULV) virions. Gn-Gc glycoprotein spikes are indicated with arrowheads. Scale bar, 50 nm. (B) Inset from (A) showing a magnified view of the membranous region of one virion. Scale bar, 15 nm. (C) A TULV virion showing higher order architecture of reconstructed TULV Gn-Gc glycoprotein spikes (gray), prepared by mapping spike complexes onto the virion lipid bilayer envelope (cyan). Zoom-in panel (bottom) reveals the higher-order glycoprotein lattice of Gn-Gc spikes. (D and E) Top (D) and side (E) views of the 16-Å-resolution TULV Gn-Gc glycoprotein spike with the fitted crystal structure of PUUV Gn. (F) Schematic of (E) with dimensions and putative density assignments annotated. See also Figures S3–S5.

Purified TULV virions are pleomorphic in shape (Figure 2A), with glycoprotein spikes encapsulating the virion (Figure 2B) and forming higher-order lattices (Figure 2C). The spike complexes extend 10 nm from the 6-nm-thick viral envelope, and the membrane-distal region of the spike consists of four lobes of globular density. These lobes form contacts with adjacent protomers of the tetramer and with stalk-like densities linking to the membrane surface. Density corresponding to the transmembrane and intraviral tails of the Gn (153 amino acids) and Gc (34 amino acids) was also partially observed (Figure S4), although was not defined well enough for fitting of the intraviral zinc-finger Gn nuclear magnetic resonance structure (Estrada et al., 2009, Estrada et al., 2011).

Consistent with the previously reported TULV structure, we observed two types of stalks linking the membrane-distal globular lobes to the virion envelope: (1) an elongated peripheral stalk that links diagonally to the membrane and cross-links with neighboring spikes and (2) a central stalk located at the center of each tetrameric spike (Figures 2D–2F). The rod-like nature and homodimeric contacts formed between adjacent peripheral stalk protomers is reminiscent to the elongated class-II fusion fold predicted for the hantaviral fusion glycoprotein (Tischler et al., 2005). Such homotypic glycoprotein contacts have also been observed for Gc glycoproteins from other bunyaviruses, including phlebo- (Dessau and Modis, 2013) and orthobunyaviruses (Bowden et al., 2013), albeit in varying oligomeric forms. We suggest that such glycoprotein cross-linking motifs may be necessary for the formation of higher-order glycoprotein lattices across genera of the *Bunyaviridae*. Together, these observations also lead us to putatively assign the elongated peripheral stalk density to the hantaviral Gc (Figure 2F). This assignment is further supported by volume analysis in Chimera (Pettersen et al., 2004), whereby each peripheral stalk density has a calculated mass of ∼51 kDa, as expected for a single protomer of the TULV Gc ectodomain (∼50 kDa).

Given the high level of sequence conservation between TULV and PUUV (78.6% identity; Figure S3) over the Gn and Gc glycoproteins and the direct relationship between sequence and structural similarity (Chothia and Lesk, 1986), we expect the TULV and PUUV glycoproteins to exhibit highly similar fold architectures. As a result, the electron microscopy (EM) structure of the TULV Gn-Gc spike constitutes a useful model for locating our PUUV Gn crystal structure on the hantaviral surface.

### Localization of PUUV Gn in the Hantavirus Spike

Computational cross-correlation-based fitting of the PUUV Gn crystal structure to the segmented cryo-ET density localized it to the four membrane-distal lobes of the spike (see Experimental Procedures). The unique density segments used in the fitting comprised two for the central stalk, two for the membrane-distal lobes, and two for the peripheral stalks. Fitting allowed identification of two alternative placements of Gn (fit A and fit B, cross-correlation coefficient 0.90–0.92; Figures S4 and S5) in both of the non-equivalent membrane-distal lobes (Figure S4, segments 1 and 2). Fits calculated for the other parts of the spike had much lower cross-correlation coefficients (<0.79) or overlapped with their symmetry related copies.

Localization of Gn to the membrane-distal lobes is consistent with previous hypotheses (Hepojoki et al., 2010) and our volume analysis, where each of the lobes corresponded to an approximate molecular mass of 38 kDa, as expected for our crystallized PUUV Gn (∼40 kDa). The membrane-distal location of Gn suggests that it is under greater immune pressure and undergoes a higher level of non-synonymous sequence variation than the more buried Gc. To investigate the selective pressures acting on PUUV Gn and Gc and to validate the localization of the fit, we analyzed sequence variation for both regions using a dataset of 25 PUUV glycoprotein sequences. The ratio of non-synonymous to synonymous nucleotide substitution (dN/dS) represents the differential effect of natural selection on these two types of mutations; lower values indicate stronger negative selection against amino acid change. As expected, the average dN/dS value was observed to be significantly lower for the Gc (dN/dS = 0.0285, 95% confidence interval [CI], [0.0249, 0.0323]) than for the Gn (dN/dS = 0.0405, 95% CI [0.0359, 0.0454]). The greater non-synonymous sequence variation of the Gn is consistent with a membrane-distal localization and supports the notion that the Gn is subjected to the selective pressure of the humoral immune response.

### Orientation of Gn in the Membrane-Distal Lobes

**Figure 3:**
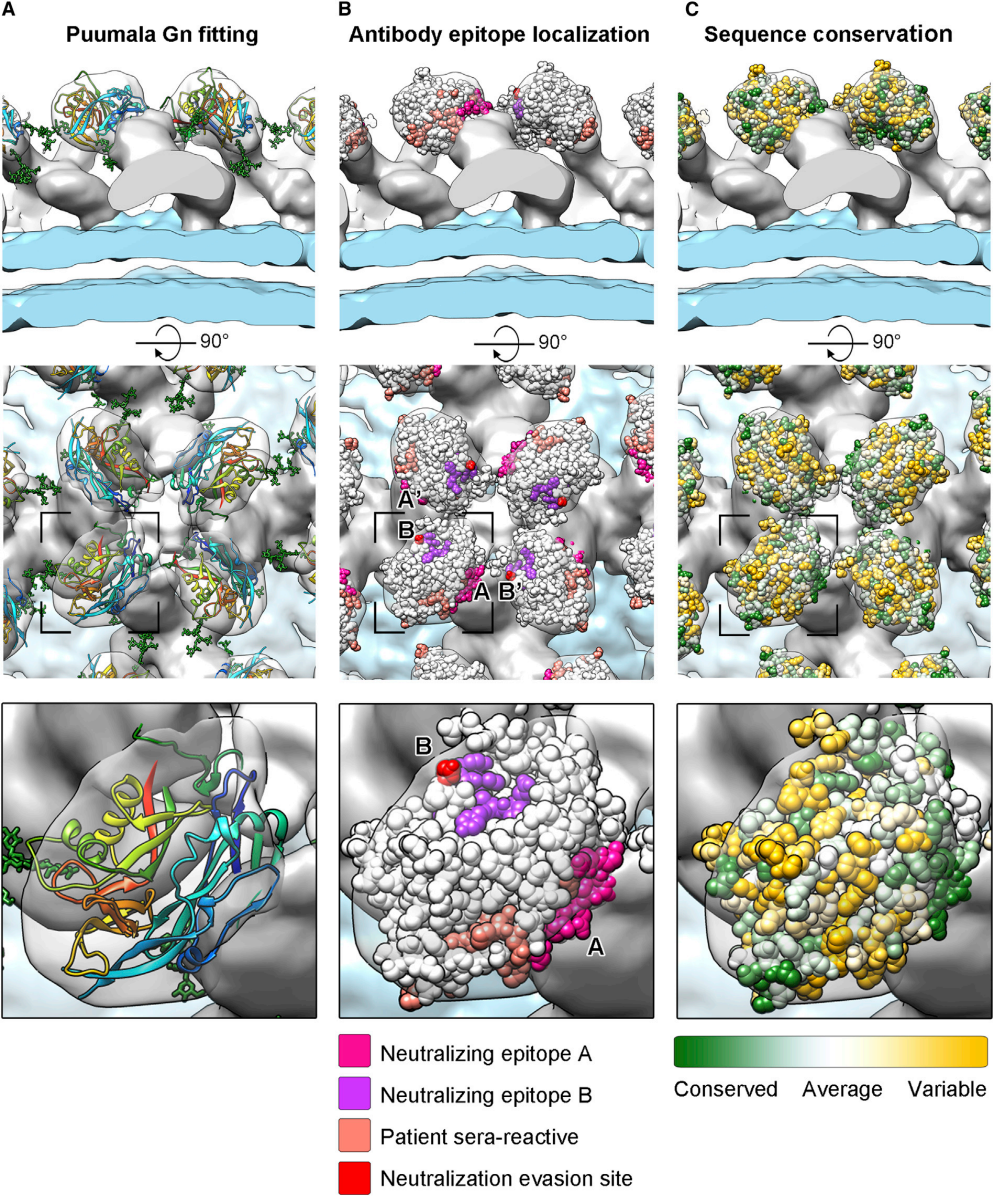
Mapping Functional Residues onto PUUV Gn Surface (A) PUUV Gn fitted into the TULV reconstruction, as in Figure 2, with zoom panel shown (bottom). (B) Mapping the antigenic surface of PUUV Gn. Predicted mAb 5A2 neutralizing epitopes are colored magenta and purple (A/A′ and B/B′, respectively). Patient sera-reactive epitopes are colored salmon. The antibody neutralization evasion site (D272V) is colored red. (C) Mapping sequence conservation onto PUUV Gn. Well-conserved (green), average (white), and variable (yellow) regions are shown. The conservation analysis was performed with Consurf (Ashkenazy et al., 2010) using the hantaviral sequences listed in the Figure S2 legend. See also Figures S2–S5.

Fitting of the PUUV Gn crystal structure into the membrane-distal part of the hantaviral spike yielded two types of solutions, A and B, with similar scoring (Figures 2, S4, and S5). As these two fittings differ in the orientation of the Gn (Figure S5), we used additional functional constraints to discern between these two possibilities. These included (1) evaluating the location of the C termini of the four Gn protomers, which contain an additional ∼120 amino acids that link to the viral membrane (Figures 3A and S5), and (2) monitoring the location of N-linked glycosylation sequons, where such post-translational modifications are not usually observed at oligomerization or protein-protein interaction interfaces (Figures 3A and S5) (Bowden et al., 2010). Analyses for both of these functional constraints support fit A, as summarized below.

#### The Gn C terminus

Our crystallized Gn ectodomain starts four amino acids after the predicted N-terminal signal sequence cleavage site and ends ∼120 amino acids prior to the predicted transmembrane region (Figure 1B). Given the fitting of the Gn globular head domain in the membrane-distal region of the hantaviral glycoprotein spike, it is likely that the C terminus of the Gn bridges toward the membrane. Indeed, in our preferred fitting of PUUV Gn tetramers (fit A), we observe that the C-terminal regions of the PUUV Gn protomers co-localize toward the center of the tetrameric spike and likely contribute to the central stalk density (Figure S5). Localization of the Gn C terminus to the central stalk is consistent with volume analysis, whereby four C-terminal stalk regions of PUUV, with a sequence-predicted molecular mass of 12.5 kDa for each of the four protomers, would be accommodated into the calculated volume of the central stalk region of the TULV glycoprotein spike (50.0 kDa).

#### N-Linked Glycans on the Gn

For our preferred PUUV Gn fitting (fit A), we observe that the N-linked glycans presented by the Gn extend from the tetrameric glycoprotein spike surface into the solvent-accessible regions between spikes (Figures 2D, 2E, and 3A). This fitting is also consistent with the projected position of a third N-linked glycosylation site, observed in the related Hantaan viral subgroup (Figure S2), which also localizes to these inter-spike regions (Figure S5).

### Antibody Epitopes on the Gn Surface

The humoral antibody response has been suggested to be sufficient for providing immunity to hantaviral infection (Schmaljohn et al., 1990), and neutralizing epitopes have been identified on both the Gn and Gc (Arikawa et al., 1989, Koch et al., 2003, Liang et al., 2003, Lundkvist et al., 1993, Lundkvist and Niklasson, 1992, Spiropoulou et al., 2003), supporting the hypothesis that both glycoproteins are antigenically exposed on the mature virion. We mapped the location of these previously identified functional epitopes onto the fitted Gn crystal structure to provide a structural context to antibody-dependent virus neutralization.

Epitopes from one such PUUV neutralizing monoclonal antibody (mAb), mAb 5A2, have been localized to three Gn sites: residues 61–71, 264–267, and 273–280 (Heiskanen et al., 1999, Heiskanen et al., 2003). In agreement with our fitting, these sites are solvent accessible (Figure 3B). However, these sites overlap with three of the five Gn-Gn interaction surfaces identified in earlier peptide scanning experiments (residues 56–73, 164–184, 257–277, 275–289, and 365–379) (Hepojoki et al., 2010) (Figures S2 and S5). We suggest that the observed overlap between antibody epitopes and proposed oligomerization interfaces may either result from mAb 5A2 targeting these interfaces or reflect a limitation of the peptide scanning technique.

In the context of the antigenic topography of PUUV Gn, the 5A2 epitope segregates onto two opposing faces of the molecule, site A (61–71) and site B (264–267 and 273–280) (Figure 3B). Due to the landscape of the Gn, the topographic distance between site A and site B (∼50 Å) is much greater than the topographic distance between site A and site B′, located within the neighboring subunit (∼25 Å). Thus, we suggest that a single 5A2 binding site may encompass two adjacent PUUV Gn protomers (sites A and B′). Interestingly, binding of 5A2 to PUUV is abrogated by a single site-directed mutation on the Gn, D272V, which has been created in vitro by directed evolution experiments (Hörling and Lundkvist, 1997). This residue locates roughly in the center of the predicted A-B′ 5A2 binding site (Figure 3B).

The targeting of multiple glycoprotein subunits of a viral glycoprotein by a single fragment antigen-binding (Fab) region is not without precedent. For example, the Fab region of monoclonal antibody PG9, which targets trimeric GP120 of HIV-1, binds at the apex of the molecule, with a single binding site extending across multiple protomeric surfaces (Julien et al., 2013). A similar phenomenon has been proposed for the anti-PUUV human antibody, 1C9, which is thought to target a mixed Gn/Gc epitope (Hepojoki et al., 2010).

Polyclonal sera derived from individuals that have been infected by PUUV have also been used to identify Gn epitopes (residues 19–33, 52–72, 79–93, and 85–99) (Heiskanen et al., 1999). Interestingly, when mapped onto the PUUV Gn surface, these epitopes overlap with one of the proposed binding sites of 5A2 (Figures 3B and S2). Furthermore, the same region of the Gn glycoprotein from Sin Nombre virus has also been observed to be immunodominant (Heiskanen et al., 1999, Jenison et al., 1994). We note the relatively high level of sequence conservation at this region of the glycoprotein (Figure 3C), which may provide a blueprint for the rational design of broad-spectrum therapeutics. Together, these data provide a unified structural model for the immunogenic hantaviral Gn.

## Discussion

Here, we determined the organization of the Gn glycoprotein on the mature hantaviral envelope. Our Gn fit is supported by several functional constraints including analysis of dN/dS, N-linked glycosylation, and the directionality of the Gn C terminus. Our PUUV Gn crystal structure was determined at pH 5.0, which is different than the pH used for the TULV virion reconstruction (pH 8.0). Although we cannot preclude the possibility that a pH change introduces subtle changes to Gn tertiary or quaternary structure, acidification had no observable effect upon Gn in solution (Figure S1), and previous biochemical analysis was not indicative of any change to the oligomeric state of the full-length protein (Acuña et al., 2015). Taken together, we propose that this fitting provides the best currently available model for the antigenic hantavirus surface.

The origin of the Gn fold is unknown. Similar to that suggested for the arenaviral α/β GP1 (Bowden et al., 2009), it is possible that the ancestral hantaviral Gn fold arose either de novo or was derived from an original host reservoir, prior to the worldwide proliferation of hantaviruses. It will be of interest to see if the hantaviral Gn fold is observed in other bunyavirus genera, as has been suggested for the class-II architecture of the cognate Gc glycoprotein (Tischler et al., 2005). Alternatively, given the diversity of glycoprotein ultra-structure assemblies observed across the family (Bowden et al., 2013), it seems equally possible that the Gn-fold architecture has diverged from a common ancestor to the extent that Gn glycoprotein structures from different genera are no longer relatable.

While the hantaviral Gc glycoprotein is arguably responsible for membrane fusion, the role of the Gn glycoprotein is unclear. It is possible that the Gn recognizes cellular receptors, such as integrins, DAF/CD55, and gC1qR, during viral attachment (Buranda et al., 2010, Choi et al., 2008, Gavrilovskaya et al., 1998, Raymond et al., 2005). Interestingly, however, the phleboviral Gc glycoprotein has also been suggested to be involved in receptor recognition (Crispin et al., 2014). Additionally, by analogy to E1−E2 complexes of alphaviruses (Li et al., 2010), the membrane-distal hantaviral Gn may be akin to the alphaviral E2 and prevent premature conformational rearrangements of the Gc fusion glycoprotein.

Hantavirus outbreaks are of special cause for concern due to the unpredictable nature of emergence and the severity of disease caused upon zoonosis to humans. Emergency health care responses to emerging hantaviral outbreaks have been severely compromised by the absence of approved therapeutics to treat infection. This combined X-ray crystallography and cryo-ET analysis provides a molecular-level description of the hantaviral surface and thus presents a rational template for targeting this deadly group of pathogens.

## Experimental Procedures

### Expression and Crystal Structure Determination of PUUV Gn

PUUV Gn (residues 29−383; GenBank: CAB43026.1) was cloned into the pHLsec vector (Aricescu et al., 2006) and transiently expressed in HEK293S cells. Following expression, cell supernatant was concentrated and dialyzed into a buffer containing 150 mM NaCl and 10 mM Tris (pH 8.0). PUUV was purified by Ni^2+^-chelated immobilized metal affinity chromatography followed by size exclusion chromatography using a Superdex 200 10/30 column (GE Healthcare). Purified PUUV Gn was crystallized, X-ray data were collected at Diamond Light Source (DLS), and the structure was solved using the SAD method (see Supplemental Experimental Procedures).

### Purification of TULV Virions

TULV (strain Moravia) was cultivated on Vero E6 cells (ATCC 94 CRL-1586), as previously described (Huiskonen et al., 2010). Three days postinfection (dpi), the growth medium was replaced to medium supplemented with 3% fetal calf serum (FCS). The virus-containing medium, collected at 5 dpi, was passed through a 0.22-μm syringe filter (Millipore) and concentrated ∼250-fold using a 100-kDa cutoff filter (Millipore), placed on top of a 0%–50% Optiprep density gradient (in 25 mM Tris and 75 mM NaCl [pH 8.0]) in a SW41 tube (Beckman Coulter), and the virus was banded by ultracentrifugation (SW41 rotor, 30,000 rpm, 5°C, 3 hr). Virus-containing fractions were pooled and concentrated using a 100-kDa cutoff filter (Millipore).

### Cryo-ET, Sub-tomogram Averaging, and Gn Fitting

A 3-μl aliquot of purified TULV and 3 μl of colloidal 10-nm gold (Aurion) were applied on a plasma-cleaned EM grid (C-flat; Protochips). Grids were blotted for 3 s followed by plunge-freezing into a mixture of liquid ethane (37%) and propane (63%) (Tivol et al., 2008).

Data were collected using a Tecnai F30 “Polara” transmission electron microscope (FEI) operated at 300 KV and at liquid nitrogen temperature. SerialEM (Mastronarde, 2005) was used to acquire tomographic tilt series on a direct electron detector (K2 Summit; Gatan) mounted behind an energy filter (QIF Quantum LS; Gatan) operated at zero-energy-loss mode (slit width, 20 eV). Movies consisting of eight frames (total exposure 1.6 s) were acquired at each tilt in electron-counting superresolution mode at a calibrated magnification of ×37,037, corresponding to a pixel size of 0.675 Å. Defocus values used were from 2.0 to 3.8 μm.

To correct for beam induced motion, frames at each tilt were aligned and averaged, and 2× binning was applied (Li et al., 2013). 3D tomograms were reconstructed using IMOD (Kremer et al., 1996). The gold beads were used as fiducial markers to align the images and were computationally removed prior to reconstruction. Contrast transfer function parameters were estimated and images corrected by phase flipping (Xiong et al., 2009). Further 2× binning was applied, resulting in the final pixel size of 2.7 Å.

Sub-tomogram averaging was carried out in Dynamo (Castaño-Díez et al., 2012) using a previously determined structure of the TULV spike (EMDB: 1704) as an initial template and following an iterative gold-standard alignment strategy (Huiskonen et al., 2014, Li et al., 2016). To reduce template bias, the initial template was filtered to 43-Å resolution (see Supplemental Experimental Procedures). The resolution of the final averaged density map was estimated by FSC using a criterion of 0.143.

The fitting of PUUV Gn into the TULV EM density map was performed with Segger (Pintilie et al., 2010) in Chimera (Pettersen et al., 2004) and is further described in Supplemental Experimental Procedures.

### Evolutionary Conservation of Amino Acid Residues

For dN/dS analysis, a dataset of 25 PUUV glycoprotein sequences were collated from GenBank. A multiple sequence alignment was generated using MUSCLE (Edgar, 2004) and average dN/dS values for Gn and Gc were estimated using the SLAC model implemented in the HYPHY package (Kosakovsky Pond and Frost, 2005, Pond et al., 2005).

Evolutionary conservation of amino acid residues was mapped onto the PUUV Gn structure using ConSurf (Ashkenazy et al., 2010) with a multiple sequence alignment of 39 hantavirus Gn sequences (generated using MUSCLE; Edgar, 2004) and a maximum likelihood phylogenetic tree (LG + G + I model; Le and Gascuel, 2008) generated with MEGA6 (Tamura et al., 2013). GenBank accession numbers are listed in the legend to Figure S2. Conservation scores were calculated using an empirical Bayesian algorithm (Mayrose et al., 2004). An LG evolutionary substitution model (Le and Gascuel, 2008) was applied.

## Author Contributions

All authors designed and performed the experiments, analyzed the data, and wrote the manuscript.

## Figures and Tables

**Table 1:** Data Collection and Refinement Statistics for PUUV Gn

Data Collection	Native PUUV Gn	K2PtCl4 (Peak)
Beamline	Diamond I03	Diamond I04
Resolution (Å)	62–2.28 (2.34–2.28)	73–3.7 (3.80–3.70)
Space group	*P*1	*P*1
Cell dimensions (Å)	*a* = 51.6, *b* = 66.8,	*a* = 49.7, *b* = 67.3,
	*c* = 77.4; α = 107.3,	*c* = 76.5; α = 105.1,
	β = 93.6, γ = 100.9	β = 96.1, γ = 100.1
Wavelength (Å)	0.9763	1.0721
Unique reflections	43,115 (3,176)	9,824 (772)
Completeness (%)	98.5 (97.6)	98.9 (99.2)
*R*_merge_^a^	0.11 (0.82)	0.17 (0.65)
*I*/σ*I*	12.1 (2.0)	12.4 (3.0)
Average redundancy	5.3 (5.0)	10.4 (6.9)
CC1/2	1.0 (0.69)	0.99 (0.86)
**Refinement**		
Resolution range (Å)	73.3–2.28 (2.34–2.28)	
Number of reflections	40,697 (2,974)	
R_factor_ (%)^b^	18.9	
R_free_ (%)^c^	21.9	
rmsd bonds (Å)	0.012	
rmsd angles (°)	1.6	
Atoms per asymmetric unit (protein/water/sugar)	5,068/338/145	
Average B factors (protein/water/sugar) (Å^2^)	49.1/44.3/73.1	
**Model quality Ramachandran plot**		
Favored regions (%)	97.5	
Allowed regions (%)	2.5

Numbers in parentheses refer to the relevant outer resolution shell. rmsd, root mean square deviation from ideal geometry. See also Figure S1.

a *R*_merge_ = Σ_hkl_ Σ_i_|*I(hkl;i)* − < *I(hkl)* > |/Σ_hkl_ Σ_i_*I(hkl;i)*, where *I(hkl;i)* is the intensity of an individual measurement and < *I(hkl)* > is the average intensity from multiple observations.

b *R*_factor_ = Σ_hkl_||*F*_obs_| – *k*|*F*_calc_||/Σ_hkl_ |*F*_obs_|.

c *R*_free_ equals the *R*_factor_ as calculated above but using against 5% of the data removed prior to refinement.

**Table 2:** Electron Cryomicroscopy Acquisition and Processing Statistics for the TULV Glycoprotein Spike Structure

Data Acquisition	TULV
Tilt range (^o^)	−45 to +45
Interval (^o^)	5
Frames per tilt	8
Total dose (e^−^/Å^2^)	∼60
Defocus^a^ (μm)	2.0−3.8
**Data processing**	
Tilt series	30
Viruses	44
Seeds	26,391
Sub-tomogram volumes^b^	5,449
Box size (pixels)	160
Pixel size (Å)	2.7
Resolution (Å)^c^	15.6

See also Figure S4.

a Positive defocus denotes underfocus.

b Number of sub-tomograms used to calculate the reconstruction.

c Resolution (Fourier shell correlation = 0.5).

## Accession Numbers

The accession number for the atomic coordinates and structure factors of PUUV Gn reported in this paper is PDB: 5FXU. The accession numbers for the EM structure of the TULV Gn-Gc complex and the fitted PUUV Gn coordinates are EMDB: EMD-3364 and PDB: 4FYN, respectively.

## Supplemental Information

Document S1. Supplemental Experimental Procedures and Figures S1–S5

Document S2. Article plus Supplemental Information
