# A Molecular-Level Account of the Antigenic Hantaviral Surface

**DOI:** 10.1016/j.celrep.2016.06.039

**Published:** 2016-06-28

**Authors:** Sai Li, Ilona Rissanen, Antra Zeltina, Jussi Hepojoki, Jayna Raghwani, Karl Harlos, Oliver G. Pybus, Juha T. Huiskonen, Thomas A. Bowden

(Cell Reports *15*, 959–967; May 3, 2016)

In the originally published version of this article, the newly reported accession number PDB: 4FYN was misreported. The number should appear as PDB: 5FYN. This has now been corrected online. The authors apologize for any confusion this error might have caused.

