# Supplementary material for: A Molecular-Level Account of the Antigenic Hantaviral Surface
Source: Cell Rep. 2016 Apr 21;15(5):959–67. doi: 10.1016/j.celrep.2016.03.082 (PMC4858563; doi:10.1016/j.celrep.2016.03.082)
Supplement: Document S1. Supplemental Experimental Procedures and Figures S1–S5 [file mmc1.pdf]

Cell Reports, Volume *15*

Supplemental Information

A Molecular-Level Account of the Antigenic Hantaviral Surface

Sai Li, Ilona Rissanen, Antra Zeltina, Jussi Hepojoki, Jayna Raghwani, Karl Harlos, Oliver G. Pybus, Juha T. Huiskonen, and Thomas A. Bowden

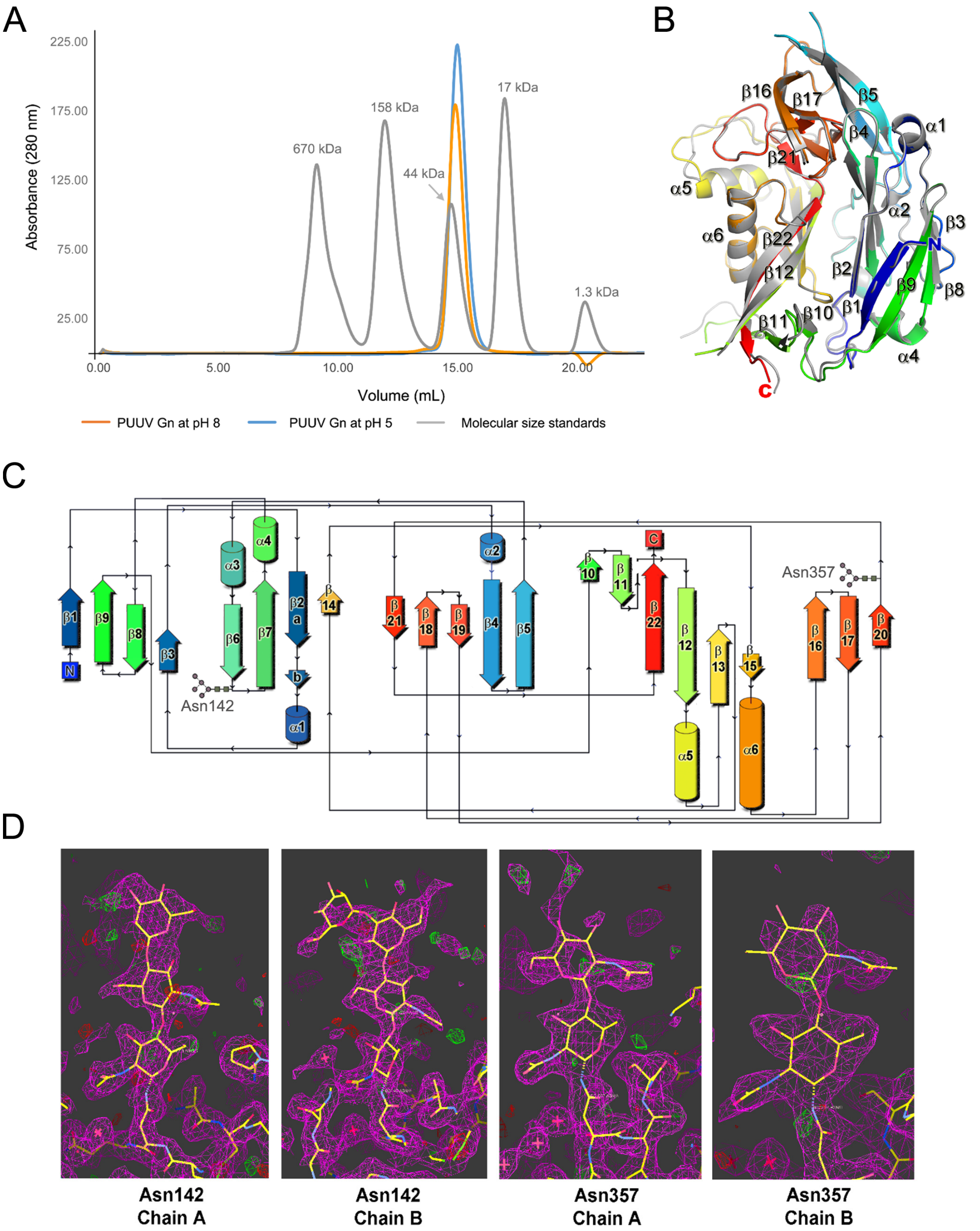

Figure S1. Structural and biochemical analysis of Puumala virus (PUUV) Gn, related to Figure 1 and Table 1. (A) Size exclusion chromatography analysis of PUUV Gn was run on a Superdex 200 10/30 column equilibrated in 150 mM NaCl and 10 mM Tris, pH 8.0 (orange curve), overlaid with a chromatogram of gel filtration standards (grey curve, Bio-Rad #151-1901) run in similar conditions. Gel filtration standard peaks correspond to thyroglobulin (670 kDa), γ-globulin (158 kDa), ovalbumin (44 kDa), myoglobin (17 kDa) and vitamin B12 (1.35 kDa). Size exclusion chromatography of PUUV Gn was also performed in 150 mM NaCl and 10 mM citrate, pH 5.0 (blue curve) and reveals that PUUV Gn remains as a putative monomeric species at pH 5.0. (B) Structural overlay of the two PUUV Gn chains observed in the asymmetric unit. Shown in cartoon

representation with one molecule colored gray and the other as a rainbow ramped from blue (N-terminus) to red (C-terminus). (C) Topology diagram of PUUV Gn ectodomain (generated with PDBsum (Laskowski, 2009)). Secondary structure elements are numbered starting from the N-terminus and shown with an arrow (β-strand) or cylinder (α-helices and the 3_{10}-type helix, α2) and are colored as in panel *B*. (D) Electron density at N-linked glycosylation sequons in the PUUV Gn crystal structure. Maximum likelihood-weighted 2*Fo*−*Fc* (pink) and *Fo*−*Fc* (green and red) electron density is shown at each N-linked glycosylation sequon. Well-ordered N-linked glycans were observed at Asn142 and Asn357 for both molecules (chains A and B) observed in the asymmetric unit of the crystal.

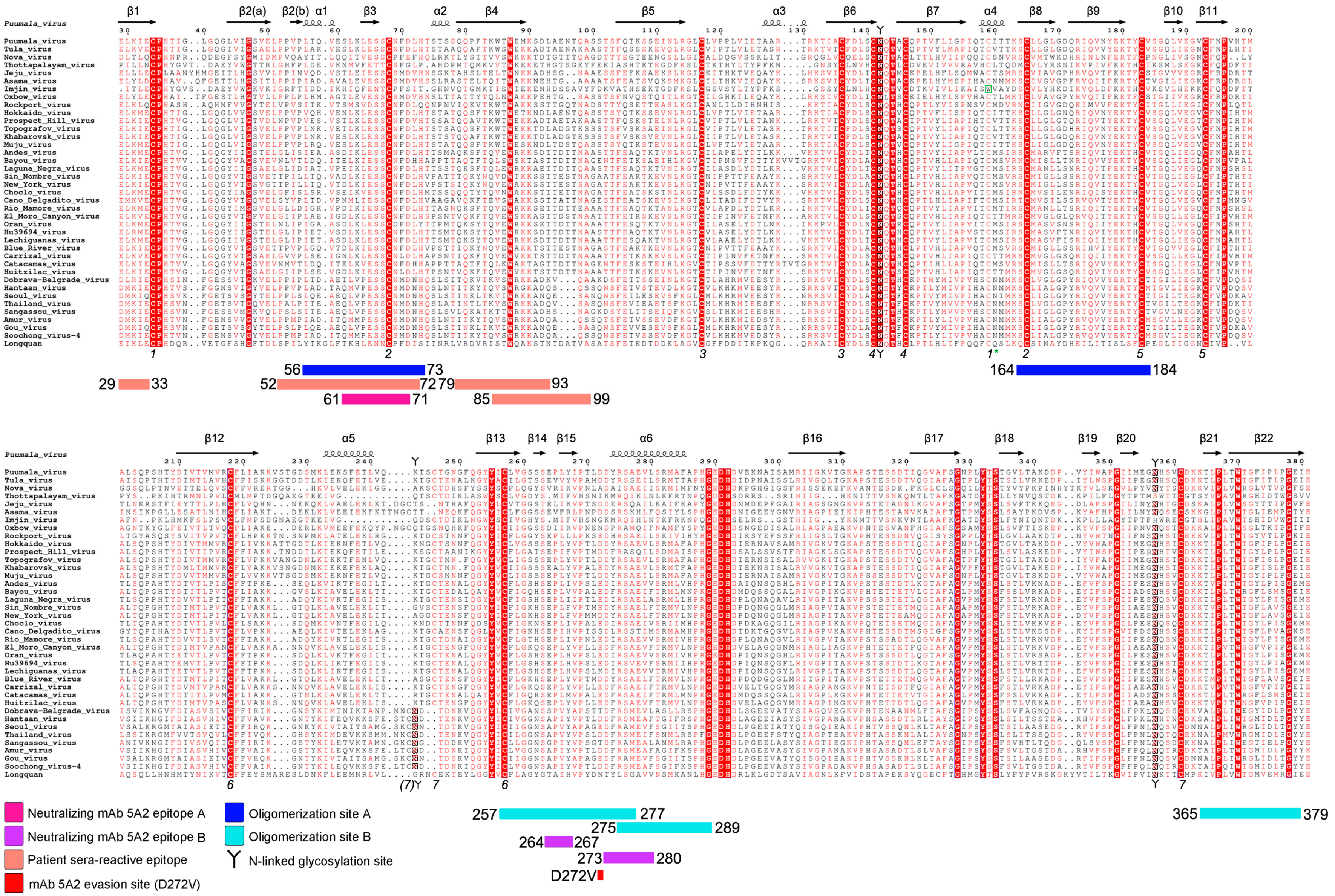
Puumala_virus
β1 β2(a) β2(b) α1 β3 α2 β4 β5 α3 β6 β7 α4 β8 β9 β10 β11
Puumala_virus
Tula_virus
Nova_virus
Thottapalayam_virus
Jeju_virus
Asama_virus
Imjin_virus
Oxbow_virus
Rockport_virus
Hokkaido_virus
Prospect_Hill_virus
Topografov_virus
Khabarovsk_virus
Muju_virus
Andes_virus
Bayou_virus
Laguna_Negra_virus
Sin_Nombre_virus
New_York_virus
Choclo_virus
Cano_Delgadito_virus
Rio_Mamore_virus
El_Moro_Canyon_virus
Oran_virus
Hu39694_virus
Lechiguanas_virus
Blue_River_virus
Carrizal_virus
Catacamas_virus
Huitzilac_virus
Dobrava-Belgrade_virus
Hantaan_virus
Seoul_virus
Thailand_virus
Sangassou_virus
Amur_virus
Gou_virus
Soochong_virus-4
Longquan
56 73
164 184
29 33
52 72 79 93
61 71
85 99
β12 α5 β13 β14 β15 α6 β16 β17 β18 β19 β20 β21 β22
257 277
275 289
264 267
273 280
D272V
365 379
Neutralizing mAb 5A2 epitope A
Neutralizing mAb 5A2 epitope B
Patient sera-reactive epitope
mAb 5A2 evasion site (D272V)
Oligomerization site A
Oligomerization site B
N-linked glycosylation site

Figure S2. Sequence alignment and mapping of functional residues included in the crystallized PUUV Gn with other hantaviral Gn glycoproteins, related to Figure 1 and 3. The alignment was prepared with ClustalW (Chenna et al., 2003) and plotted with ESPRIPT (Gouet et al., 1999). Residues which are highlighted in red are fully conserved, residues which are colored red are partially conserved, and residues which are colored black are not conserved. Amino acids corresponding to predicted N-linked glycosylation sites are outlined in black and marked with black trees. Secondary structure from the PUUV crystal structure was mapped above the alignment, where helices, including the 3_{10}-type helix, α2, are shown as spirals and β-strands are shown as arrows. Cysteines participating in disulfide bonds are numbered, in italics, underneath the alignment, and the single instance (Imjin virus) where the cysteine pattern is not conserved is annotated with a green box. Cysteines likely participating in disulfide bond seven occupy slightly different positions in the sequences of different viruses, and are thus denoted by (7) and 7. The epitopes illustrated in main Figure 3 are presented as bars below the alignment as follows: predicted neutralizing and patient sera-reactive epitopes (Heiskanen et al., 1999) are shown in magenta and purple (mAb 5A2 epitopes A and B, respectively) and salmon (patient sera-reactive epitopes) and the mAb 5A2 neutralization evasion site (D272V) (Horling and Lundkvist, 1997) is shown in red. In the context of our preferred Gn_{ecto} fitting into the Tula reconstruction (main Figure 3), these antigenic regions are solvent exposed. Interestingly, the PUUV antigenic epitopes around the mAb 5A2 epitope A also overlap with the previously identified immunodominant epitope in Sin Nombre virus (SNV residues 59-89) (Jenison et al., 1994).The two sets of oligomerization sites identified on the Gn surface (Hepojoki et al., 2010) are annotated dark blue (site A) and cyan (site B). Residues 164-184 and 365-379 are buried within the putative oligomerization interface, while residues 56-73 (site A) and residues 257-289 (site B) exhibit a greater degree of solvent accessibility. GenBank accession numbers for the used sequences are as follows: Puumala virus CAB43026.1; Hokkaido virus BAM13711.1; Muju virus AGE45110.1; Khabarovsk virus AIL25323.1; Topografov virus CAB42098.1; Tula virus NP_942586.1; Prospect Hill virus CAA38922.1, Huitzilac virus BAK08526.1; El Moro Canyon virus AAA87198.1; Blue River virus AAC03793.1; Sin Nombre virus AFV71282.1; Bayou virus AAA61690.1; Cano Delgadito virus ABB88646.1; Andes virus AAO86638.1; Oran virus AAB87910.1; Laguna Negra virus AAB87603.1; Rio Mamore virus ACU46022.1; Choclo virus ABB90558.1; Hantaan virus BAA05012.1; Amur virus AGE13901.1; Seoul virus ABW05099.1; Thailand virus AAA16239.1; Dobrava-Belgrade virus ADP21266.1; Sangassou virus AEZ02947.1; Asama virus ACI28506.1; Oxbow virus ACT68338.1; Jeju virus AEX56232.1; Nova virus AKE37996.1; Thottapalayam virus AIF28800.1; Imjin virus AIF28793.1; Rockport virus AEA11485.1; Longquan virus AGI62348.1, Soochong virus-4 AAY56327.1; Gou virus AGC97139.1; Hu39694 virus AAB87909.1; Lechiguanas virus AAB87908.1; Catacamas virus ABA39272.1; New York virus AAC54561.1; Carrizal virus BAK08523.1.

		1	2	3	4	5	6	7	8	9	10	11	12	13	14	15	16	17	18	19	20	21	22	23	24	25	26	27	28	29	30	31	32	33	34	35	36	37	38	39
1	Thottapalayam virus																																							
2	Imjin_virus	70.2																																						
3	Longquan virus	38.9	39.7																																					
4	Nova virus	42.5	41.9	44.9																																				
5	Rockport virus	43.1	41.9	41.7	48.9																																			
6	Prospect Hill virus	42.7	42.5	41.6	47.3	61.6																																		
7	Tula virus	42.7	44.1	40.3	47.4	63.3	79.7																																	
8	Puumala virus	42.6	42.5	40.9	46.4	63.2	75.6	78.6																																
9	Hokkaido virus	43.0	42.5	40.9	46.0	62.2	77.0	80.0	91.3																															
10	Muju virus	42.6	42.4	40.6	46.5	63.1	76.4	79.4	89.4	92.4																														
11	Topografov virus	43.5	42.9	41.1	46.9	62.3	77.2	81.1	82.6	83.9	84.6																													
12	Khabarovsk virus	43.2	42.6	41.9	46.5	62.4	78.4	81.3	82.9	84.2	85.4	90.4																												
13	Cano Delgadito virus	43.1	42.5	41.0	48.3	62.2	64.5	66.0	64.5	64.9	65.2	66.0	65.7																											
14	Choclo virus	43.7	43.7	40.2	46.8	61.4	65.8	66.4	64.4	65.1	65.1	65.5	66.1	76.4																										
15	Andes virus	43.4	44.9	40.6	46.4	62.5	66.5	67.7	66.1	66.2	66.4	67.7	66.8	73.8	82.4																									
16	Oran virus	43.0	44.2	40.6	46.8	61.9	66.3	67.9	65.6	66.0	66.5	67.3	66.8	73.7	82.3	91.9																								
17	Hu39694 virus	43.2	44.3	40.2	46.7	61.7	66.6	68.0	65.8	66.2	66.7	67.8	67.4	73.5	81.9	92.0	94.1																							
18	Lechiguanas virus	43.5	44.8	40.8	47.2	62.3	66.7	68.3	65.8	66.3	66.7	67.8	67.2	73.7	83.0	93.0	95.4	95.5																						
19	Laguna Negra virus	43.2	44.0	39.8	46.1	61.1	65.9	67.4	65.4	65.9	66.9	67.1	66.9	73.9	81.6	86.4	86.3	86.3	86.6																					
20	Rio Mamore virus	43.7	44.9	41.1	46.5	60.9	66.6	68.2	66.2	66.5	66.8	67.8	67.3	74.7	83.1	87.4	88.3	88.0	88.9	91.2																				
21	Bayou virus	42.8	43.1	40.1	46.2	61.6	66.1	68.2	64.6	64.6	65.6	67.5	67.4	75.8	77.4	75.2	75.7	75.5	75.6	75.6	75.8																			
22	Catacamas virus	42.7	43.2	39.6	46.1	61.7	65.5	68.3	64.0	64.2	65.3	67.0	67.1	75.3	76.8	74.9	75.2	75.0	75.5	75.5	75.0	93.6																		
23	Blue River virus	42.3	43.0	39.9	46.7	61.9	67.5	69.1	67.7	67.8	68.2	68.0	68.5	76.5	77.3	76.9	77.0	76.9	77.2	76.2	76.4	79.8	79.5																	
24	Sin Nombre virus	42.7	43.1	41.0	46.7	62.6	67.3	69.1	67.7	67.3	67.5	67.4	68.1	77.2	78.0	77.5	77.5	77.5	78.1	76.6	77.1	80.5	80.3	93.8																
25	New York virus	42.4	43.3	40.5	46.7	62.0	67.5	69.2	67.7	67.4	67.8	67.7	68.0	76.1	76.9	76.7	77.2	77.4	77.6	76.2	76.4	79.5	79.7	93.8	95.5															
26	El Moro Canyon virus	43.5	42.9	40.4	46.5	61.3	66.5	68.5	66.3	66.7	67.0	67.8	67.5	73.6	74.8	73.3	72.9	73.3	73.7	74.2	74.4	76.6	76.2	78.8	79.5	78.8														
27	Carrizal virus	43.2	43.0	40.5	47.3	62.8	67.4	68.9	67.1	66.8	67.7	67.9	68.5	74.6	76.5	74.4	75.2	74.7	75.7	75.0	75.0	77.9	77.5	79.9	80.4	79.6	90.1													
28	Huitzilac virus	43.2	42.4	40.7	47.2	62.7	67.1	69.1	67.5	67.0	67.9	69.1	68.6	74.9	75.9	75.3	75.0	74.9	75.3	75.3	75.1	78.3	77.5	81.0	81.9	80.7	91.0	93.0												
29	Hantaan virus	43.5	43.9	39.5	45.5	53.9	54.3	55.8	54.7	55.2	55.5	55.4	55.8	55.7	54.4	55.0	55.1	55.3	55.8	54.8	55.5	55.4	55.7	55.7	55.1	54.7	56.5	56.5	56.9											
30	Amur virus	43.5	44.4	39.9	45.3	53.1	54.7	54.9	55.0	55.9	55.0	55.3	55.9	55.1	54.4	55.2	55.4	55.4	55.7	55.4	56.4	55.3	55.2	56.0	55.2	54.9	55.8	56.0	56.4	91.7										
31	Soochong virus-4	43.4	44.4	39.6	45.4	53.3	54.3	55.0	54.8	55.9	54.9	54.9	56.1	55.7	54.8	55.0	55.3	55.5	55.7	55.6	56.4	55.5	55.7	56.3	55.6	55.0	56.3	56.5	56.7	92.2	97.2									
32	Thailand virus	43.4	44.6	39.3	46.4	53.0	54.1	54.6	54.0	54.6	55.5	54.4	55.0	54.7	53.7	53.9	54.5	54.3	54.7	53.6	54.7	54.6	54.9	54.0	53.9	53.9	55.8	56.4	56.2	77.1	76.2	76.9								
33	Seoul virus	43.0	43.4	38.7	45.6	53.6	53.8	54.4	54.1	53.8	54.4	54.5	55.5	54.3	53.3	54.3	54.7	54.4	54.8	54.1	54.3	54.3	54.3	53.3	53.3	53.1	54.4	55.3	55.3	76.4	75.8	76.2	81.5							
34	Gou virus	43.2	43.5	39.0	45.3	53.2	53.8	54.2	54.1	54.2	54.3	54.7	55.7	54.5	53.2	54.3	55.0	54.5	54.9	54.2	54.6	54.0	53.7	53.3	53.2	53.1	54.8	55.6	55.4	76.9	76.2	76.8	82.2	96.7						
35	Dobrava-Belgrade virus	43.8	44.4	39.6	45.7	53.4	53.9	55.2	55.0	55.0	54.6	54.6	55.3	54.9	54.5	54.7	54.4	54.7	55.1	54.4	54.9	55.4	55.2	55.0	55.2	54.7	55.2	55.7	56.0	77.0	76.4	76.7	76.3	76.6	76.4					
36	Sangassou virus	43.7	43.8	39.4	46.0	53.9	53.9	54.4	54.3	55.0	54.4	54.5	54.5	53.9	53.5	54.2	54.2	54.2	54.6	54.2	55.0	55.3	55.0	54.1	53.6	53.8	54.3	54.6	55.1	76.2	76.0	76.3	77.3	77.7	77.9	80.5				
37	Jeju virus	41.9	43.3	39.9	45.3	52.0	52.4	51.7	51.9	53.1	53.3	54.2	53.9	53.9	53.2	51.6	52.0	52.2	51.9	51.2	52.1	53.2	53.4	53.7	53.2	53.5	54.7	53.8	54.2	57.7	57.3	57.2	56.8	55.8	56.0	56.8	57.4			
38	Asama virus	41.9	43.3	39.8	46.3	51.5	51.7	52.6	52.4	52.6	51.9	52.3	52.4	54.7	53.8	53.7	53.8	53.6	53.6	53.8	53.7	53.2	53.8	54.7	54.1	54.5	54.5	54.1	54.9	59.6	60.0	60.5	59.9	59.1	59.6	59.9	58.6	64.5		
39	Oxbow virus	42.0	42.5	39.8	46.1	51.2	52.0	52.1	52.0	52.0	51.6	53.2	52.8	52.6	53.6	51.9	52.3	52.1	52.0	51.4	52.0	53.5	53.4	53.4	53.3	53.1	52.7	52.7	52.8	59.8	59.3	59.4	58.6	56.9	57.2	58.6	57.7	61.3	65.8	

Figure S3. Percent identity matrix of hantavirus glycoprotein precursor sequences, related to Figures 2 and 3. Colours indicate high (green) to low (red) amino acid identity values. Percent identity matrix and the underlying multiple sequence alignment were created by MUSCLE (Edgar, 2004). Names of the viruses borne by rodents of the family *Cricetidae* are shown in blue (subfamilies *Sigmodontinae* and *Neotominae*) and green (subfamily *Arvicolinae*). In gray, viruses that are carried by Old World mice and rats (family *Muridae*). Soricomorph- and bat-borne hantaviruses are shown in red and pink, respectively. The GenBank accession numbers used in the analysis are listed in Supplementary Figure Legend S2.

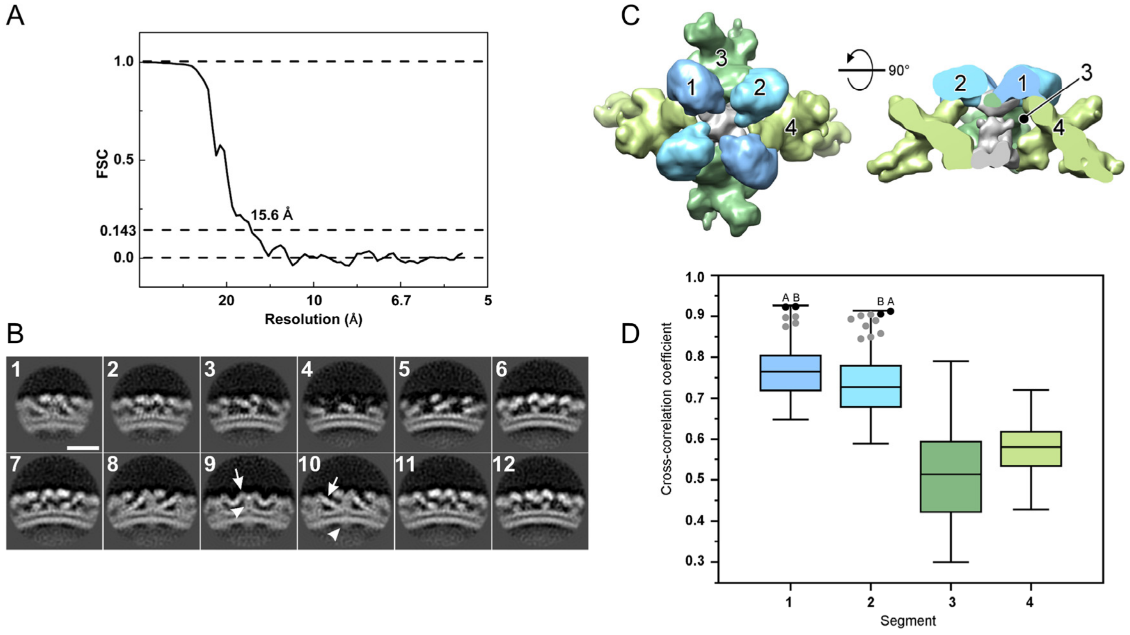

Figure S4. Analysis of the Tula virus (TULV) spike structure and fitting of PUUV Gn into the EM density, related to Figure 2, 3, and Table 2. (A) Fourier Shell Correlation (FSC) curve. To prevent overfitting during refinement, 'gold-standard' refinement was implemented by splitting the dataset into two halves and refining the two sets independently. At the end of each iteration, the FSC of the two averaged structures was computed, giving a resolution using criteria of 0.143, which was set as the low-pass for the next iteration. The final resolution of the structure was assessed by FSC analysis, giving 15.6 Å. (B) Slices through the TULV spike structure (1-12). Slices (1.35 nm thickness) through the volume from the side of the spike, nominal to the membrane. Putative regions corresponding to the Gc ectodomain (arrow, slice 10), Gc cytoplasmic domain (arrow head, slice 10), Gn membrane-distal domain (arrow, slice 9), and Gn membrane-proximal domain (arrow head, slice 9) are annotated. Scale bar, 14 nm. (C) Analysis of PUUV Gn ectodomain crystal structure fitting into TULV EM density. Density extracted from the TULV spike reconstruction is shown from the top (left) and side (right). Different density segments used in the fitting of the PUUV Gn ectodomain crystal structure are rendered in different colors and numbered. (D) A box-and-whisker plot showing the interquartile ranges of the cross-correlation coefficients between the crystal structure and four different segments (1–4) of the EM density. Cross-correlation values were calculated for 1000 evenly rotated fits (box plots). Segments 1 and 2, corresponding to the membrane-distal lobes of the spike tetramer, gave consistently higher cross-correlation values than segments 3 and 4, corresponding to the peripheral stalks of the spike. Two segments corresponding to the central stalk of the spike (rendered gray in panel *C*) were excluded from the analysis due to clashes between the fitted structure and its symmetry related copy. The top scoring optimized unique fits against segments 1 and 2 are denoted with gray and black circles. The two top fits (black circles labeled A and B) had very similar cross-correlation values (segment 1: A=0.922, B=0.924; segment 2: A=0.912, B=0.904) and correspond to the two alternative fitting solutions described in the maintext and in Figure S5.

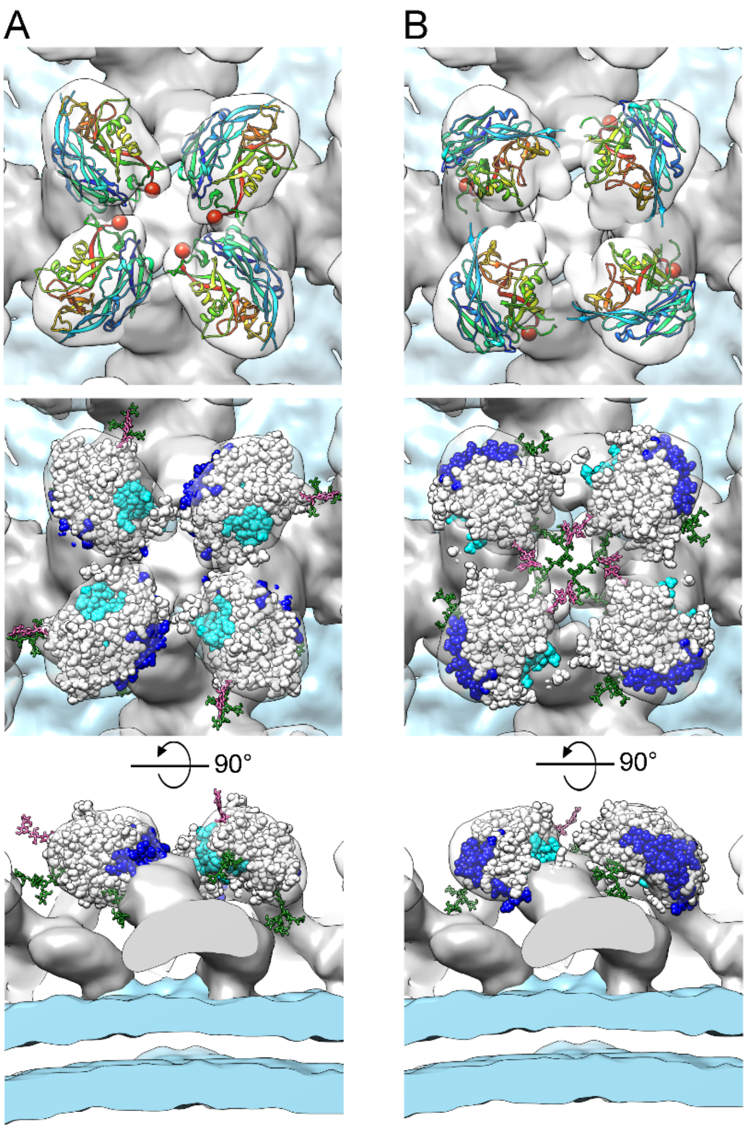

Figure S5. Fitting of PUUV Gn into the 16 Å TULV reconstruction yielded two types of possible solutions (panels *A* and *B*), related to Figure 2 and 3. Gn-Gc glycoprotein spikes are colored gray and the virion lipid bilayer envelope is colored light blue. The membrane-bound glycoprotein spike of TULV with the crystal structure of PUUV Gn fitted into the membrane distal lobes is shown. In the topmost panels, fitted PUUV Gn crystal structures are shown as ribbons colored as a rainbow ramped from blue (N-terminus) to red (C-terminus). Red spheres indicate the position of the C-termini for each fitted Gn. The two types of solutions generated by Segger (see **Supplementary Experimental Procedures**) prompted mapping of N-linked glycans and known oligomerization sites on the PUUV Gn surface as restraints to assess the validity of the fits. Sites of homotypic Gn oligomerisation (Hepojoki et al., 2010) are colored blue and cyan (as defined in **Figure S2**). As the hantaviral glycans presented on Gn and Gc are endoglycosidase H sensitive (Shi and Elliott, 2004), high mannose-type glycans (from PDB 2WAH (Crispin et al., 2009)) are modeled as green sticks at N-linked sequons (Asn142 and Asn357). Both of these sites were observed to be occupied in the crystal structure. An additional putative N-linked glycosylation site present within the Hantaan virus subgroup (defined in **Figure S2** and **S3**) was modeled onto PUUV Gn as pink sticks at the putative N-linked glycosylation sequon (Asn235 in

Hantaan virus and Lys243 in PUUV). As described in the main text, the following observations (*i*-*ii*) provide confidence to our selected fitting: (*i*) The chosen fit (panel *A*) localizes the Gn to the tetrameric, membrane-distal lobes of the spike such that C-terminal transmembrane anchor regions co-localize and point towards the virion envelope. In the rejected fit (panel *B*), the C-termini of the Gn are pointed outwards from the spike towards inter-glycoprotein spike solvent regions. (*ii*) For our selected PUUV Gn fitting (panel *A*), we observe that N-linked glycans on the Gn extend from the tetrameric glycoprotein spike surface into inter-spike solvent accessible regions. In contrast, these N-linked glycans sterically clash with neighboring glycans within the same spike in the alternative fit (panel *B*).

SUPPLEMENTARY EXPERIMENTAL PROCEDURES

Crystal structure determination and refinement

PUUV Gn was concentrated to 6.0 mg per mL in 150 mM NaCl and 10 mM Tris pH 8.0 and crystallized after 15 days using the vapour diffusion method (Walter et al., 2005) by mixing 100 nl protein plus 100 nl of a reservoir containing 30% w/v polyethylene glycol 6000 and 0.1 M citrate buffer pH 5.0. PUUV Gn crystals were cryo-protected by immersion into a solution containing the crystallization reservoir plus 25% ethylene glycol and then rapidly cryo-cooled by immersion into liquid nitrogen.

Native data were collected at Diamond Light Source (DLS) beamline I03 to a resolution of 2.3 Å (λ = 0.9763). For phase determination, a crystal was soaked for 2 h with ~10 mM potassium tetrachloroplatinate (II), prior to cryo-protection. Diffraction data of the Pt-soaked crystal were collected at DLS beamline I04 at the L3 edge for platinum (λ = 1.0721) to a resolution of 3.7 Å. Diffraction images were indexed, integrated, and scaled with XIA2 (Winter, 2010). Five percent of reflections were randomly set aside to calculate the R_{free}. The single wavelength anomalous diffraction (SAD) method was employed for phase determination and heavy atom positions were identified, refined, and density modified in autoSHARP (de La Fortelle and Bricogne, 1997). The resulting low-resolution (3.7 Å) electron density map was used for initial building of the resulting two molecules in the asymmetric unit and a nearly complete model was built automatically using the high-resolution native data in autoBUILD (Terwilliger et al., 2008).

The structure of the Gn was refined using Refmac5 in the CCP4 suite (1994; Winn et al., 2003) and included iterative restrained refinement with automatically generated local non-crystallographic symmetry restraints and TLS (translation-libration-screw rotation) parameterization. Coot (Emsley and Cowtan, 2004) was used for manual rebuilding and MolProbity (Davis et al., 2007) was used to validate the model.

Sub-tomogram averaging

To locate spikes on the virions, the membrane of the virions was first modeled using either a sphere or an ellipsoid in Dynamo (Castano-Diez et al., 2012) *tomoview*, where evenly distributed and oriented pseudo-particles (denoted here as 'seeds') were created (Huiskonen et al., 2014). Seeds were defined with a seed-to-seed spacing of 28 pixels, resulting in an average of 600 seeds for each virus. 26,391 subtomograms were extracted from 44 virions at the seed locations into 160×160×160 voxels boxes. In the first stage of the iterative refinement scheme (Huiskonen et al., 2014), extracted densities were cylindrically averaged for each virus. The best average was used as an initial template to align all the seeds relative to the virion membrane allowing shifts only orthogonal to the plane of the membrane. At the end of each iteration, a custom post-processing Dynamo function was used to adjust the shift of each seed further by a weighting scheme taking the shifts of its neighbors into consideration as well. The contribution of each neighbor was weighted by a Gaussian function taking into account its distance to the seed in question, similarly to motion correction in single particle processing (Scheres, 2014). In the second stage, a previously determined TULV spike structure (EMDB-1704) (Huiskonen et al., 2010) was used as a template to locate the spikes on the virions. A shell-like mask encompassing a spike and some contribution from the adjacent four neighbors and two-fold symmetry was applied. The resolution was restricted to 43 Å for the first iteration and shifts of 28 pixels were allowed lateral to the membrane. At the end

of each iteration, a custom post-processing function was used to remove all seeds that were too close to each other except for the one with the best cross-correlation. In the third stage, all six parameters (three location coordinates and three Euler angles) were allowed to change simultaneously. The coordinates and orientations of particles were plotted and 1% of the particles whose coordinates and orientations deviated from the geometry of the virus were removed. Also, 10% of the particles with the bottom cross-correlation scores were removed. The resolution of the results was estimated by Fourier shell correlation (FSC) using a criterion of 0.143 (**Figure S4**).

To prevent over-refinement (correlation of noise instead of signal) during the refinement strategy described above, a custom post-processing function was designed to carry out 'gold-standard' refinements since the second stage, where the data is split into two independent sets and refinements were carried out independently. At the end of each iteration, the FSC of the two averages was calculated using a criterion of 0.143. After subtracting one shell from the corresponding value, the estimated resolution was used as low-pass filter for the next iteration.

Fitting PUUV Gn structure into the TULV reconstruction

For fitting of PUUV Gn, the TULV EM density map was segmented with Segger (Pintilie et al., 2010) in Chimera (Pettersen et al., 2004). The surface threshold was determined by matching the volume of one $(Gn–Gc)_4$ ectodomain spike to the theoretical volume corresponding to the calculated mass of the $(Gn–Gc)_4$ (416 kDa; constant protein density 0.81 Da/Å^3). The PUUV Gn crystal structure was converted to density by low pass filtering it to 16-Å resolution and 1,000 evenly rotated fits were considered for each segment (**Figure S4**). All fits in the central stalk region overlapped with their symmetry related copies (clash score >0.5) and were discarded from further analysis. Fitting to the membrane-distal lobes of the spike resulted in higher cross-correlation values than fitting to the other parts of the spike complex (**Figure S4**). From the pool of 1,000 evenly rotated fits, the best scoring unique fits were generated by using the 'optimize fits' option of Segger. Redundant fits less than 5 Å or 3 degrees apart were discarded.

SUPPLEMENTARY REFERENCES

(1994). The CCP4 suite: programs for protein crystallography. Acta Crystallogr D Biol Crystallogr *50*, 760-763.

Castano-Diez, D., Kudryashev, M., Arheit, M., and Stahlberg, H. (2012). Dynamo: a flexible, user-friendly development tool for subtomogram averaging of cryo-EM data in high-performance computing environments. J Struct Biol *178*, 139-151.

Chenna, R., Sugawara, H., Koike, T., Lopez, R., Gibson, T.J., Higgins, D.G., and Thompson, J.D. (2003). Multiple sequence alignment with the Clustal series of programs. Nucleic Acids Res *31*, 3497-3500.

Crispin, M., Bowden, T.A., Coles, C.H., Harlos, K., Aricescu, A.R., Harvey, D.J., Stuart, D.I., and Jones, E.Y. (2009). Carbohydrate and domain architecture of an immature antibody glycoform exhibiting enhanced effector functions. J Mol Biol *387*, 1061-1066.

Davis, I.W., Leaver-Fay, A., Chen, V.B., Block, J.N., Kapral, G.J., Wang, X., Murray, L.W., Arendall, W.B., 3rd, Snoeyink, J., Richardson, J.S., *et al.* (2007). MolProbity: all-atom contacts and structure validation for proteins and nucleic acids. Nucleic Acids Res *35*, W375-383.

de La Fortelle, E., and Bricogne, G. (1997). Maximum-likelihood heavy-atom parameter refinement for multiple isomorphous replacement and multiwavelength anomalous diffraction methods. In Methods in Enzymology, C.C.a.S. RM, ed. (New York: Academic Press), pp. 472-494.

Edgar, R.C. (2004). MUSCLE: multiple sequence alignment with high accuracy and high throughput. Nucleic Acids Res *32*, 1792-1797.

Emsley, P., and Cowtan, K. (2004). Coot: model-building tools for molecular graphics. Acta Crystallogr D Biol Crystallogr *60*, 2126-2132.

Gouet, P., Courcelle, E., Stuart, D.I., and Metoz, F. (1999). ESPript: analysis of multiple sequence alignments in PostScript. Bioinformatics *15*, 305-308.

Heiskanen, T., Lundkvist, A., Soliymani, R., Koivunen, E., Vaheri, A., and Lankinen, H. (1999). Phage-displayed peptides mimicking the discontinuous neutralization sites of puumala Hantavirus envelope glycoproteins. Virology *262*, 321-332.

Hepojoki, J., Strandin, T., Vaheri, A., and Lankinen, H. (2010). Interactions and oligomerization of hantavirus glycoproteins. Journal of virology *84*, 227-242.

Horling, J., and Lundkvist, A. (1997). Single amino acid substitutions in Puumala virus envelope glycoproteins G1 and G2 eliminate important neutralization epitopes. Virus Res *48*, 89-100.

Huiskonen, J.T., Hepojoki, J., Laurinmaki, P., Vaheri, A., Lankinen, H., Butcher, S.J., and Grunewald, K. (2010). Electron cryotomography of Tula hantavirus suggests a unique assembly paradigm for enveloped viruses. J Virol *84*, 4889-4897.

Huiskonen, J.T., Parsy, M.L., Li, S., Bitto, D., Renner, M., and Bowden, T.A. (2014). Averaging of viral envelope glycoprotein spikes from electron cryotomography reconstructions using Jsubtomo. J Vis Exp, e51714.

Jenison, S., Yamada, T., Morris, C., Anderson, B., Torrez-Martinez, N., Keller, N., and Hjelle, B. (1994). Characterization of human antibody responses to four corners hantavirus infections among patients with hantavirus pulmonary syndrome. Journal of virology *68*, 3000-3006.

Laskowski, R.A. (2009). PDBsum new things. Nucleic Acids Res *37*, D355-359.

Pettersen, E.F., Goddard, T.D., Huang, C.C., Couch, G.S., Greenblatt, D.M., Meng, E.C., and Ferrin, T.E. (2004). UCSF Chimera--a visualization system for exploratory research and analysis. J Comput Chem *25*, 1605-1612.

Pintilie, G.D., Zhang, J., Goddard, T.D., Chiu, W., and Gossard, D.C. (2010). Quantitative analysis of cryo-EM density map segmentation by watershed and scale-space filtering, and fitting of structures by alignment to regions. J Struct Biol *170*, 427-438.

Scheres, S.H. (2014). Beam-induced motion correction for sub-megadalton cryo-EM particles. Elife *3*, e03665.

Shi, X., and Elliott, R.M. (2004). Analysis of N-linked glycosylation of hantaan virus glycoproteins and the role of oligosaccharide side chains in protein folding and intracellular trafficking. J Virol *78*, 5414-5422.

Terwilliger, T.C., Grosse-Kunstleve, R.W., Afonine, P.V., Moriarty, N.W., Zwart, P.H., Hung, L.W., Read, R.J., and Adams, P.D. (2008). Iterative model building, structure refinement and density modification with the PHENIX AutoBuild wizard. Acta Crystallogr D Biol Crystallogr *64*, 61-69.

Walter, T.S., Diprose, J.M., Mayo, C.J., Siebold, C., Pickford, M.G., Carter, L., Sutton, G.C., Berrow, N.S., Brown, J., Berry, I.M.*, et al.* (2005). A procedure for setting up high-throughput nanolitre crystallization experiments. Crystallization workflow for initial screening, automated storage, imaging and optimization. Acta Cryst D *61*, 651-657.

Winn, M.D., Murshudov, G.N., and Papiz, M.Z. (2003). Macromolecular TLS refinement in REFMAC at moderate resolutions. Methods Enzymol *374*, 300-321.

Winter, G. (2010). xia2: an expert system for macromolecular crystallography data reduction. Journal of Applied Crystallography *43*, 186-190.
