# Supplementary material for: A Molecular-Level Account of the Antigenic Hantaviral Surface
Source: Cell Rep. 2016 Apr 21;15(5):959–67. doi: 10.1016/j.celrep.2016.03.082 (PMC4858563; doi:10.1016/j.celrep.2016.03.082)
Supplement: Document S2. Article plus Supplemental Information [file mmc2.pdf]

Report

Cell Reports

A Molecular-Level Account of the Antigenic Hantaviral Surface

Graphical Abstract

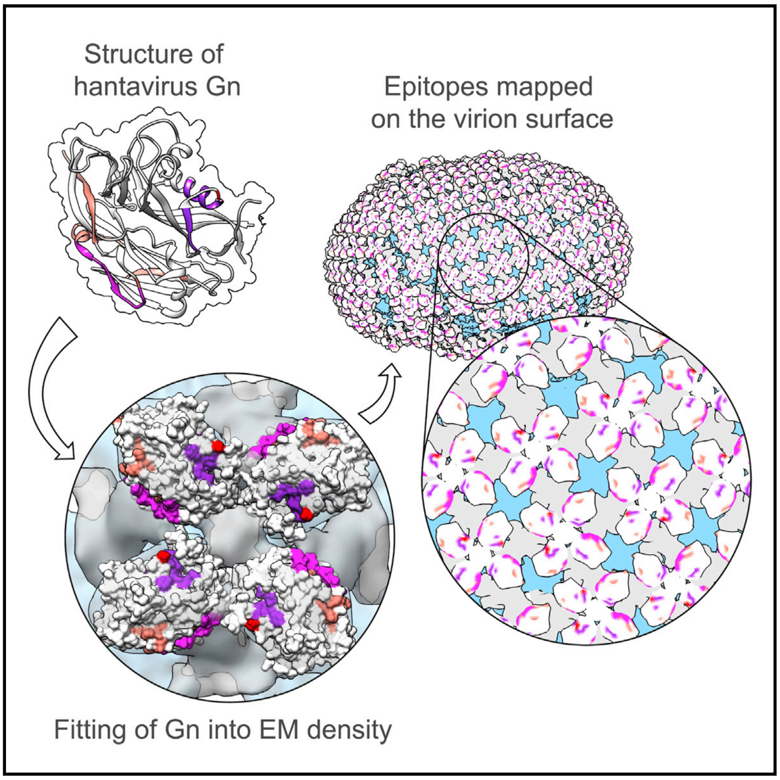

Highlights

- We describe the high-resolution crystal structure of a hantaviral Gn ectodomain
- Electron cryotomography analysis reveals the ultrastructure of Gn-Gc assembly
- X-ray fitting and mapping analysis reveals the antigenic hantavirus surface
- The Gn fold is likely to be widely conserved across this group of viruses

Authors

Sai Li, Ilona Rissanen, Antra Zeltina, ..., Oliver G. Pybus, Juha T. Huiskonen, Thomas A. Bowden

Correspondence

juha.huiskonen@strubi.ox.ac.uk (J.T.H.), thomas.bowden@strubi.ox.ac.uk (T.A.B.)

In Brief

Li et al. combine X-ray crystallography with electron cryotomography and sub-tomogram averaging to determine the organization of hantaviral glycoproteins on the mature virion envelope. This work provides a molecular-level description of the antigenic surface for a group of widespread and deadly viral pathogens.

Accession Numbers

5FXU
EMD-3364
4FYN

 ©2016 The Authors
http://dx.doi.org/10.1016/j.celrep.2016.03.082

Cell Reports
Report

OPEN ACCESS
CellPress

A Molecular-Level Account of the Antigenic Hantaviral Surface

Sai Li,[1] Ilona Rissanen,[1] Antra Zeltina,[1] Jussi Hepojoki,[2] Jayna Raghwani,[3] Karl Harlos,[1] Oliver G. Pybus,[3] Juha T. Huiskonen,[1,*] and Thomas A. Bowden[1,*]
[1]Division of Structural Biology, Wellcome Trust Centre for Human Genetics, University of Oxford, Roosevelt Drive, Oxford OX3 7BN, UK
[2]Department of Virology, Haartman Institute, University of Helsinki, 00014 Helsinki, Finland
[3]Department of Zoology, University of Oxford, South Parks Road, Oxford OX1 3PS, UK
*Correspondence: juha.huiskonen@strubi.ox.ac.uk (J.T.H.), thomas.bowden@strubi.ox.ac.uk (T.A.B.)
http://dx.doi.org/10.1016/j.celrep.2016.03.082

SUMMARY

Hantaviruses, a geographically diverse group of zoonotic pathogens, initiate cell infection through the concerted action of Gn and Gc viral surface glycoproteins. Here, we describe the high-resolution crystal structure of the antigenic ectodomain of Gn from Puumala hantavirus (PUUV), a causative agent of hemorrhagic fever with renal syndrome. Fitting of PUUV Gn into an electron cryomicroscopy reconstruction of intact Gn-Gc spike complexes from the closely related but non-pathogenic Tula hantavirus localized Gn tetramers to the membrane-distal surface of the virion. The accuracy of the fitting was corroborated by epitope mapping and genetic analysis of available PUUV sequences. Interestingly, Gn exhibits greater non-synonymous sequence diversity than the less accessible Gc, supporting a role of the host humoral immune response in exerting selective pressure on the virus surface. The fold of PUUV Gn is likely to be widely conserved across hantaviruses.

INTRODUCTION

Hantaviruses, from the family *Bunyaviridae*, constitute a genus of human pathogens with a near-worldwide distribution (Jonsson et al., 2010). These viruses chronically and asymptomatically infect rodents, shrews, moles, and bats. Cross-species transmission to humans, primarily via aerosolized animal excreta, can lead to severe diseases (Jonsson et al., 2010; Lee and Johnson, 1982; Nuzum et al., 1988). Clinical symptoms of hantavirus infection usually manifest two to three weeks following initial exposure and lead to either hantavirus pulmonary syndrome (HPS) or hemorrhagic fever with renal syndrome (HFRS) (Lednicky, 2003). The case-mortality rates typically range from 0.1 to 10 % for HFRS to up to 40% for HPS (Vaheri et al., 2013).

Hantaviruses have a lipid-bilayer envelope, and their negative-sense RNA genome is divided into S, M, and L segments. The ~1,150-amino-acid glycoprotein precursor is encoded by the M segment (Schmaljohn et al., 1987) and is co-translationally cleaved by the cellular signal peptidase complex at the conserved “WAASA” sequence (Löber et al., 2001) into two structural glycoprotein components, Gn (~70 kDa) and Gc (~55 kDa). Low resolution three-dimensional (3D) structures of Tula (TULV) and Hantaan virus spike complexes, derived by electron cryomicroscopy studies and combined with biochemical analysis, revealed that Gn and Gc form square-shaped oligomeric complexes on the virion envelope (Battisti et al., 2011; Hepojoki et al., 2010; Huiskonen et al., 2010).

Similar to the Gc from Rift Valley fever virus (genus *Phlebovirus*), another *Bunyaviridae* family member (Dessau and Modis, 2013), the hantaviral Gc is expected to form a class-II membrane fusion protein fold (Tischler et al., 2005). The fold of the Gn ectodomain, on the other hand, is unknown. Following an initial interaction between a cell-surface receptor and the hantaviral Gn-Gc complex, the virus is endocytosed and fusion of the cellular and viral membranes is thought to occur via a pH-dependent process (Acuña et al., 2015; Jin et al., 2002). Several cell-surface glycoproteins, including integrins, the decay-accelerating factor (DAF/CD55), and complement receptor gC1qR, have been suggested as viral entry receptors (Buranda et al., 2010; Choi et al., 2008; Gavrilovskaya et al., 1998; Raymond et al., 2005).

We determined the crystal structure of the Gn ectodomain from Puumala virus (PUUV), a hantavirus endemic in common vole populations throughout Eurasia and responsible for nephropathia epidemica, a mild form of HFRS. Using electron cryotomography (cryo-ET), we resolved the structure of the envelope glycoprotein spike complex from the closely related apathogenic Tula virus (TULV) to 16 Å resolution. This facilitated fitting of the Gn to the four membrane-distal lobes of the spike, a placement corroborated by estimation of synonymous and non-synonymous nucleotide substitutions in PUUV sequences and mapping of previous biochemical analyses on the structure. Combined with antibody epitope mapping, these data provide a detailed description of the antigenic hantaviral surface.

RESULTS

Expression of the PUUV Gn ectodomain

Similar to other hantaviruses (Schmaljohn et al., 1987), PUUV Gn encodes a signal sequence (residues 1–24) (Petersen et al., 2011), an N-terminal ectodomain (residues 25–504), a predicted transmembrane region (residues 505–526) (Krogh et al., 2001), and a C-terminal cytoplasmic domain (residues 527–658). To

This is an open access article under the CC BY license (http://creativecommons.org/licenses/by/4.0/).

Table 1. Data Collection and Refinement Statistics for PUUV Gn

Data Collection	Native PUUV Gn	K2PtCl4 (Peak)
Beamline	Diamond I03	Diamond I04
Resolution (Å)	62–2.28 (2.34–2.28)	73–3.7 (3.80–3.70)
Space group	*P*1	*P*1
Cell dimensions (Å)	a = 51.6, b = 66.8,	a = 49.7, b = 67.3,
	c = 77.4; α = 107.3,	c = 76.5; α = 105.1,
	β = 93.6, γ = 100.9	β = 96.1, γ = 100.1
Wavelength (Å)	0.9763	1.0721
Unique reflections	43,115 (3,176)	9,824 (772)
Completeness (%)	98.5 (97.6)	98.9 (99.2)
R_{merge}[a]	0.11 (0.82)	0.17 (0.65)
$I/\sigma I$	12.1 (2.0)	12.4 (3.0)
Average redundancy	5.3 (5.0)	10.4 (6.9)
CC1/2	1.0 (0.69)	0.99 (0.86)
Refinement		
Resolution range (Å)	73.3–2.28 (2.34–2.28)	
Number of reflections	40,697 (2,974)	
R_{factor} (%)[b]	18.9	
R_{free} (%)[c]	21.9	
rmsd bonds (Å)	0.012	
rmsd angles (°)	1.6	
Atoms per asymmetric unit (protein/water/sugar)	5,068/338/145	
Average B factors (protein/water/sugar) (Å^2)	49.1/44.3/73.1	
Model quality Ramachandran plot		
Favored regions (%)	97.5	
Allowed regions (%)	2.5	

Numbers in parentheses refer to the relevant outer resolution shell. rmsd, root mean square deviation from ideal geometry. See also Figure S1.
[a]$R_{merge} = \Sigma_{hkl} \Sigma_i |I(hkl;i) - <I(hkl)>| / \Sigma_{hkl} \Sigma_i I(hkl;i)$, where $I(hkl;i)$ is the intensity of an individual measurement and $<I(hkl)>$ is the average intensity from multiple observations.
[b]$R_{factor} = \Sigma_{hkl} ||F_{obs}| - k|F_{calc}|| / \Sigma_{hkl} |F_{obs}|$.
[c]R_{free} equals the R_{factor} as calculated above but using against 5% of the data removed prior to refinement.

facilitate soluble protein expression, a PUUV Gn construct (residues 29−383) was truncated by ~120 residues prior to the C-terminal transmembrane helix and transiently expressed in HEK293S cells. As observed by size-exclusion chromatography in both neutral (pH 8.0) and acidic (pH 5.0) conditions (Figure S1), PUUV Gn is a monomer in solution, consistent with the hypothesis that residues 450 onward contribute to tetramer formation (Hepojoki et al., 2010).

Structure of PUUV Gn

The crystal structure of PUUV Gn was determined to 2.3 Å resolution using the single-wavelength anomalous diffraction (SAD) method (Table 1). PUUV Gn forms an α/β fold (~40 kDa), consisting of five α helices, a 3_{10} helix, and twenty-two β strands. The β strands assemble to form five β sheets, which associate together by the formation of a β sandwich (Figure 1). The two molecules of PUUV Gn present in the crystal asymmetric unit are almost identical, with differences being limited to solvent-accessible loops (0.7 Å root mean square deviation in equivalent Cα positions over 327 residues; Figure S1). For both molecules in the asymmetric unit, three loops (residues 92−102, 204−208, and 292−300) were not clearly visible in the electron density, and it is likely that these residues are either naturally flexible or require an associated protein, such as neighboring Gn/Gc protomers, to impose order. No higher order oligomerization was detected from the crystallographic packing, supporting the hypothesis that the Gc glycoprotein and/or C-terminal regions of the Gn may, in part, be required for tetramer formation (Hepojoki et al., 2010). The PUUV Gn fold is stabilized by seven intra-domain disulfide bonds, a pattern well-conserved among hantaviruses (Figure S2). This, together with the comparatively high level of sequence conservation across rodent-borne hantaviruses (>50%; Figure S3), suggests that the observed fold is a defining feature of the genus.

The presence of N-linked, predominantly high-mannose glycosylation on the hantaviral Gn is another shared feature across the genus (Figure S2) (Johansson et al., 2004; Shi and Elliott, 2004). The PUUV Gn sequence exhibits N-linked glycosylation sequons at Asn142, Asn357, and Asn409 (which was not included in the crystallized construct). Electron density was observed at both Asn142 and Asn357 (Figure S1), with the glycans extending away from the protein surface. It is likely that the well-ordered nature of these moieties is induced by stabilizing contacts with adjacent molecules in the crystal. These data suggest that both N-linked glycan sites are occupied on PUUV virions.

Structure of the Hantaviral Surface

Apathogenic TULV is one of the closest known relatives to PUUV and a model for hantavirus ultrastructure (Huiskonen et al., 2010). We set out to study the architecture of Gn/Gc glycoprotein complexes to facilitate localization of our Gn crystal structure on the virion. Combining established techniques in cryo-ET and sub-tomogram averaging (Huiskonen et al., 2014) with direct-detector technology (Bammes et al., 2012), we improved the resolution of the TULV Gn/Gc spike structure from 36 Å (Huiskonen et al., 2010) to 16 Å (Table 2).

Purified TULV virions are pleomorphic in shape (Figure 2A), with glycoprotein spikes encapsulating the virion (Figure 2B) and forming higher-order lattices (Figure 2C). The spike complexes extend 10 nm from the 6-nm-thick viral envelope, and the membrane-distal region of the spike consists of four lobes of globular density. These lobes form contacts with adjacent protomers of the tetramer and with stalk-like densities linking to the membrane surface. Density corresponding to the transmembrane and intraviral tails of the Gn (153 amino acids) and Gc (34 amino acids) was also partially observed (Figure S4), although was not defined well enough for fitting of the intraviral zinc-finger Gn nuclear magnetic resonance structure (Estrada et al., 2009, 2011).

Consistent with the previously reported TULV structure, we observed two types of stalks linking the membrane-distal globular lobes to the virion envelope: (1) an elongated peripheral stalk

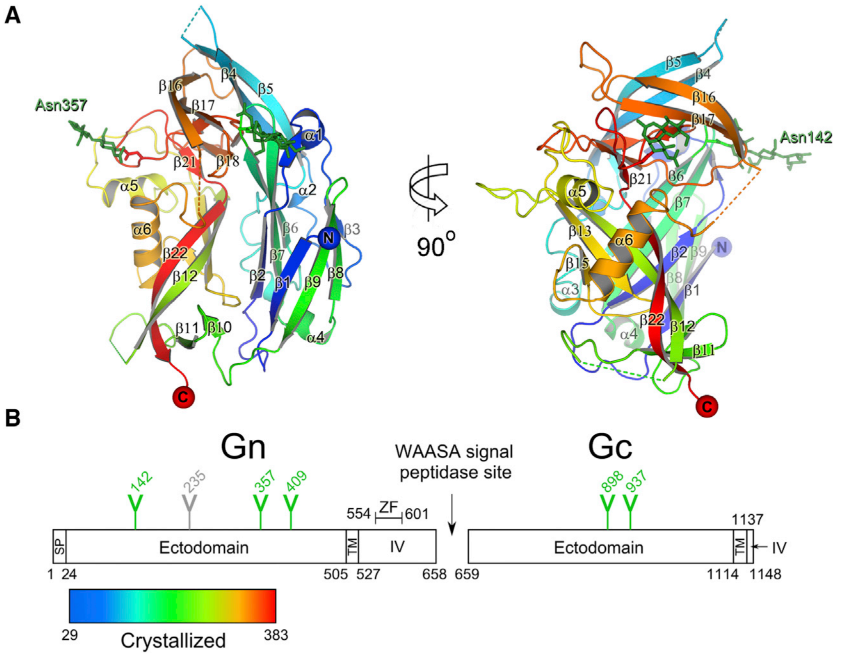

Figure 1. Crystal Structure of the Puumala Gn Ectodomain

(A) A ribbon representation of Puumala (PUUV) Gn colored from blue (N terminus) to red (C terminus). N-linked glycans are shown as green sticks.

(B) Domain schematic of PUUV glycoprotein precursor with the signal peptide (SP), ectodomain, transmembrane domain (TM), intravirion domain (IV), zinc finger (ZF), and WAASA signal peptidase cleavage site shown (produced with DOG; Ren et al., 2009). Y-shaped symbols designate N-linked glycosylation sites. The location of the additional putative N-linked glycosylation site at Asn235 in Hantaan virus (Lys243 in PUUV) is indicated in gray.

See also Figures S1 and S2.

that links diagonally to the membrane and cross-links with neighboring spikes and (2) a central stalk located at the center of each tetrameric spike (Figures 2D–2F). The rod-like nature and homodimeric contacts formed between adjacent peripheral stalk protomers is reminiscent to the elongated class-II fusion fold predicted for the hantaviral fusion glycoprotein (Tischler et al., 2005). Such homotypic glycoprotein contacts have also been observed for Gc glycoproteins from other bunyaviruses, including phlebo- (Dessau and Modis, 2013) and orthobunyaviruses (Bowden et al., 2013), albeit in varying oligomeric forms. We suggest that such glycoprotein cross-linking motifs may be necessary for the formation of higher-order glycoprotein lattices across genera of the *Bunyaviridae*. Together, these observations also lead us to putatively assign the elongated peripheral stalk density to the hantaviral Gc (Figure 2F). This assignment is further supported by volume analysis in Chimera (Pettersen et al., 2004), whereby each peripheral stalk density has a calculated mass of ~51 kDa, as expected for a single protomer of the TULV Gc ectodomain (~50 kDa).

Given the high level of sequence conservation between TULV and PUUV (78.6% identity; Figure S3) over the Gn and Gc glycoproteins and the direct relationship between sequence and structural similarity (Chothia and Lesk, 1986), we expect the TULV and PUUV glycoproteins to exhibit highly similar fold architectures. As a result, the electron microscopy (EM) structure of the TULV Gn-Gc spike constitutes a useful model for locating our PUUV Gn crystal structure on the hantaviral surface.

Localization of PUUV Gn in the Hantavirus Spike

Computational cross-correlation-based fitting of the PUUV Gn crystal structure to the segmented cryo-ET density localized it to the four membrane-distal lobes of the spike (see Experimental Procedures). The unique density segments used in the fitting comprised two for the central stalk, two for the membrane-distal lobes, and two for the peripheral stalks. Fitting allowed identification of two alternative placements of Gn (fit A and fit B, cross-correlation coefficient 0.90–0.92; Figures S4 and S5) in both of the non-equivalent membrane-distal lobes (Figure S4, segments 1 and 2). Fits calculated for the other parts of the spike had much lower cross-correlation coefficients (<0.79) or overlapped with their symmetry related copies.

Localization of Gn to the membrane-distal lobes is consistent with previous hypotheses (Hepojoki et al., 2010) and our volume analysis, where each of the lobes corresponded to an approximate molecular mass of 38 kDa, as expected for our crystallized PUUV Gn (~40 kDa). The membrane-distal location of Gn suggests that it is under greater immune pressure and undergoes a higher level of non-synonymous sequence variation than the more buried Gc. To investigate the selective pressures acting on PUUV Gn and Gc and to validate the localization of the fit, we analyzed sequence variation for both regions using a dataset of 25 PUUV glycoprotein sequences. The ratio of non-synonymous to synonymous nucleotide substitution (dN/dS) represents the differential effect of natural selection on these two types of mutations; lower values indicate stronger negative selection against amino acid change. As expected, the average dN/dS value was observed to be significantly lower for the Gc (dN/dS = 0.0285, 95% confidence interval [CI], [0.0249, 0.0323]) than for the Gn (dN/dS = 0.0405, 95% CI [0.0359, 0.0454]). The greater non-synonymous sequence variation of the Gn is consistent with a membrane-distal localization and supports the notion that the Gn is subjected to the selective pressure of the humoral immune response.

Orientation of Gn in the Membrane-Distal Lobes

Fitting of the PUUV Gn crystal structure into the membrane-distal part of the hantaviral spike yielded two types of solutions, A and B, with similar scoring (Figures 2, S4, and S5). As these two fittings differ in the orientation of the Gn (Figure S5), we used additional functional constraints to discern between these two possibilities. These included (1) evaluating the location of the

Table 2. Electron Cryomicroscopy Acquisition and Processing Statistics for the TULV Glycoprotein Spike Structure

Data Acquisition	TULV
Tilt range (°)	−45 to +45
Interval (°)	5
Frames per tilt	8
Total dose ($e^{-}/Å^2$)	~60
Defocus[a] (μm)	2.0−3.8
Data processing	
Tilt series	30
Viruses	44
Seeds	26,391
Sub-tomogram volumes[b]	5,449
Box size (pixels)	160
Pixel size (Å)	2.7
Resolution (Å)[c]	15.6

See also Figure S4.
[a]Positive defocus denotes underfocus.
[b]Number of sub-tomograms used to calculate the reconstruction.
[c]Resolution (Fourier shell correlation = 0.5).

C termini of the four Gn protomers, which contain an additional ~120 amino acids that link to the viral membrane (Figures 3A and S5), and (2) monitoring the location of N-linked glycosylation sequons, where such post-translational modifications are not usually observed at oligomerization or protein-protein interaction interfaces (Figures 3A and S5) (Bowden et al., 2010). Analyses for both of these functional constraints support fit A, as summarized below.

The Gn C terminus

Our crystallized Gn ectodomain starts four amino acids after the predicted N-terminal signal sequence cleavage site and ends ~120 amino acids prior to the predicted transmembrane region (Figure 1B). Given the fitting of the Gn globular head domain in the membrane-distal region of the hantaviral glycoprotein spike, it is likely that the C terminus of the Gn bridges toward the membrane. Indeed, in our preferred fitting of PUUV Gn tetramers (fit A), we observe that the C-terminal regions of the PUUV Gn protomers co-localize toward the center of the tetrameric spike and likely contribute to the central stalk density (Figure S5). Localization of the Gn C terminus to the central stalk is consistent with volume analysis, whereby four C-terminal stalk regions of PUUV, with a sequence-predicted molecular mass of 12.5 kDa for each of the four protomers, would be accommodated into the calculated volume of the central stalk region of the TULV glycoprotein spike (50.0 kDa).

N-Linked Glycans on the Gn

For our preferred PUUV Gn fitting (fit A), we observe that the N-linked glycans presented by the Gn extend from the tetrameric glycoprotein spike surface into the solvent-accessible regions between spikes (Figures 2D, 2E, and 3A). This fitting is also consistent with the projected position of a third N-linked glycosylation site, observed in the related Hantaan viral subgroup (Figure S2), which also localizes to these inter-spike regions (Figure S5).

Antibody Epitopes on the Gn Surface

The humoral antibody response has been suggested to be sufficient for providing immunity to hantaviral infection (Schmaljohn et al., 1990), and neutralizing epitopes have been identified on both the Gn and Gc (Arikawa et al., 1989; Koch et al., 2003; Liang et al., 2003; Lundkvist et al., 1993; Lundkvist and Niklasson, 1992; Spiropoulou et al., 2003), supporting the hypothesis that both glycoproteins are antigenically exposed on the mature virion. We mapped the location of these previously identified functional epitopes onto the fitted Gn crystal structure to provide a structural context to antibody-dependent virus neutralization.

Epitopes from one such PUUV neutralizing monoclonal antibody (mAb), mAb 5A2, have been localized to three Gn sites: residues 61–71, 264–267, and 273–280 (Heiskanen et al., 1999, 2003). In agreement with our fitting, these sites are solvent accessible (Figure 3B). However, these sites overlap with three of the five Gn-Gn interaction surfaces identified in earlier peptide scanning experiments (residues 56–73, 164–184, 257–277, 275–289, and 365–379) (Hepojoki et al., 2010) (Figures S2 and S5). We suggest that the observed overlap between antibody epitopes and proposed oligomerization interfaces may either result from mAb 5A2 targeting these interfaces or reflect a limitation of the peptide scanning technique.

In the context of the antigenic topography of PUUV Gn, the 5A2 epitope segregates onto two opposing faces of the molecule, site A (61–71) and site B (264–267 and 273–280) (Figure 3B). Due to the landscape of the Gn, the topographic distance between site A and site B (~50 Å) is much greater than the topographic distance between site A and site B′, located within the neighboring subunit (~25 Å). Thus, we suggest that a single 5A2 binding site may encompass two adjacent PUUV Gn protomers (sites A and B′). Interestingly, binding of 5A2 to PUUV is abrogated by a single site-directed mutation on the Gn, D272V, which has been created in vitro by directed evolution experiments (Hörling and Lundkvist, 1997). This residue locates roughly in the center of the predicted A-B′ 5A2 binding site (Figure 3B).

The targeting of multiple glycoprotein subunits of a viral glycoprotein by a single fragment antigen-binding (Fab) region is not without precedent. For example, the Fab region of monoclonal antibody PG9, which targets trimeric GP120 of HIV-1, binds at the apex of the molecule, with a single binding site extending across multiple protomeric surfaces (Julien et al., 2013). A similar phenomenon has been proposed for the anti-PUUV human antibody, 1C9, which is thought to target a mixed Gn/Gc epitope (Hepojoki et al., 2010).

Polyclonal sera derived from individuals that have been infected by PUUV have also been used to identify Gn epitopes (residues 19–33, 52–72, 79–93, and 85–99) (Heiskanen et al., 1999). Interestingly, when mapped onto the PUUV Gn surface, these epitopes overlap with one of the proposed binding sites of 5A2 (Figures 3B and S2). Furthermore, the same region of the Gn glycoprotein from Sin Nombre virus has also been observed to be immunodominant (Heiskanen et al., 1999; Jenison et al., 1994). We note the relatively high level of sequence conservation at this region of the glycoprotein (Figure 3C), which may provide a blueprint for the rational design of broad-spectrum therapeutics. Together, these data provide a unified structural model for the immunogenic hantaviral Gn.

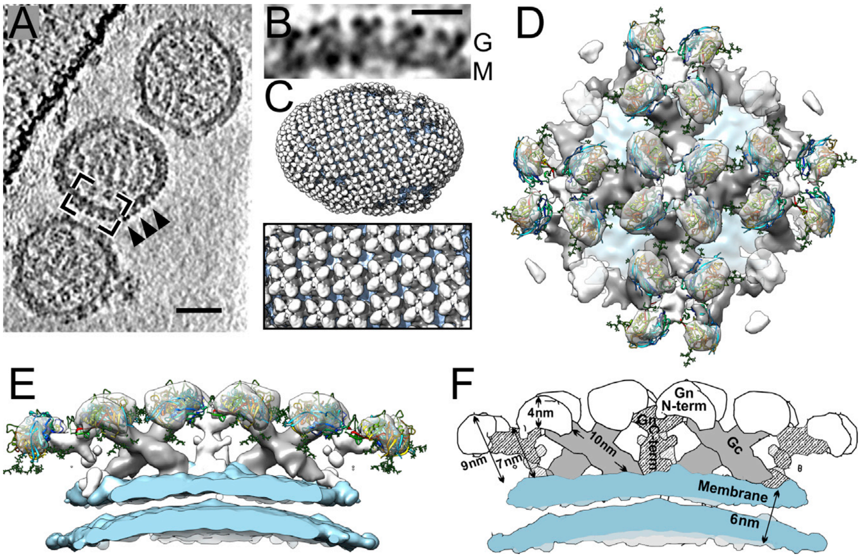

Figure 2. Organization of Tula Virion

(A) Low-pass filtered computational section from a tomographic reconstruction of three Tula virus (TULV) virions. Gn-Gc glycoprotein spikes are indicated with arrowheads. Scale bar, 50 nm.

(B) Inset from (A) showing a magnified view of the membranous region of one virion. Scale bar, 15 nm.

(C) A TULV virion showing higher order architecture of reconstructed TULV Gn-Gc glycoprotein spikes (gray), prepared by mapping spike complexes onto the virion lipid bilayer envelope (cyan). Zoom-in panel (bottom) reveals the higher-order glycoprotein lattice of Gn-Gc spikes.

(D and E) Top (D) and side (E) views of the 16-Å-resolution TULV Gn-Gc glycoprotein spike with the fitted crystal structure of PUUV Gn.

(F) Schematic of (E) with dimensions and putative density assignments annotated.

See also Figures S3–S5.

DISCUSSION

Here, we determined the organization of the Gn glycoprotein on the mature hantaviral envelope. Our Gn fit is supported by several functional constraints including analysis of dN/dS, N-linked glycosylation, and the directionality of the Gn C terminus. Our PUUV Gn crystal structure was determined at pH 5.0, which is different than the pH used for the TULV virion reconstruction (pH 8.0). Although we cannot preclude the possibility that a pH change introduces subtle changes to Gn tertiary or quaternary structure, acidification had no observable effect upon Gn in solution (Figure S1), and previous biochemical analysis was not indicative of any change to the oligomeric state of the full-length protein (Acuña et al., 2015). Taken together, we propose that this fitting provides the best currently available model for the antigenic hantavirus surface.

The origin of the Gn fold is unknown. Similar to that suggested for the arenaviral α/β GP1 (Bowden et al., 2009), it is possible that the ancestral hantaviral Gn fold arose either de novo or was derived from an original host reservoir, prior to the worldwide proliferation of hantaviruses. It will be of interest to see if the hantaviral Gn fold is observed in other bunyavirus genera, as has been suggested for the class-II architecture of the cognate Gc glycoprotein (Tischler et al., 2005). Alternatively, given the diversity of glycoprotein ultra-structure assemblies observed across the family (Bowden et al., 2013), it seems equally possible that the Gn-fold architecture has diverged from a common ancestor to the extent that Gn glycoprotein structures from different genera are no longer relatable.

While the hantaviral Gc glycoprotein is arguably responsible for membrane fusion, the role of the Gn glycoprotein is unclear. It is possible that the Gn recognizes cellular receptors, such as integrins, DAF/CD55, and gC1qR, during viral attachment (Buranda et al., 2010; Choi et al., 2008; Gavrilovskaya et al., 1998; Raymond et al., 2005). Interestingly, however, the phleboviral Gc glycoprotein has also been suggested to be involved in receptor recognition (Crispin et al., 2014). Additionally, by analogy to E1−E2 complexes of alphaviruses (Li et al., 2010), the membrane-distal hantaviral Gn may be akin to the alphaviral E2 and prevent premature conformational rearrangements of the Gc fusion glycoprotein.

Hantavirus outbreaks are of special cause for concern due to the unpredictable nature of emergence and the severity of disease caused upon zoonosis to humans. Emergency health care responses to emerging hantaviral outbreaks have been severely compromised by the absence of approved therapeutics to treat infection. This combined X-ray crystallography and cryo-ET analysis provides a molecular-level description of the hantaviral surface and thus presents a rational template for targeting this deadly group of pathogens.

EXPERIMENTAL PROCEDURES

Expression and Crystal Structure Determination of PUUV Gn

PUUV Gn (residues 29−383; GenBank: CAB43026.1) was cloned into the pHLsec vector (Aricescu et al., 2006) and transiently expressed in HEK293S cells. Following expression, cell supernatant was concentrated and dialyzed into a buffer containing 150 mM NaCl and 10 mM Tris (pH 8.0). PUUV was purified by Ni^{2+}-chelated immobilized metal affinity chromatography followed by size exclusion chromatography using a Superdex 200 10/30 column (GE Healthcare). Purified PUUV Gn was crystallized, X-ray data were collected at Diamond Light Source (DLS), and the structure was solved using the SAD method (see Supplemental Experimental Procedures).

Purification of TULV Virions

TULV (strain Moravia) was cultivated on Vero E6 cells (ATCC 94 CRL-1586), as previously described (Huiskonen et al., 2010). Three days postinfection (dpi), the growth medium was replaced to medium supplemented with 3% fetal calf serum (FCS). The virus-containing medium, collected at 5 dpi, was passed through a 0.22-μm syringe filter (Millipore) and concentrated ~250-fold using a 100-kDa cutoff filter (Millipore), placed on top of a 0%–50% Optiprep density gradient (in 25 mM Tris and 75 mM NaCl [pH 8.0]) in a SW41 tube (Beckman Coulter), and the virus was banded by ultracentrifugation (SW41 rotor, 30,000 rpm, 5°C, 3 hr). Virus-containing fractions were pooled and concentrated using a 100-kDa cutoff filter (Millipore).

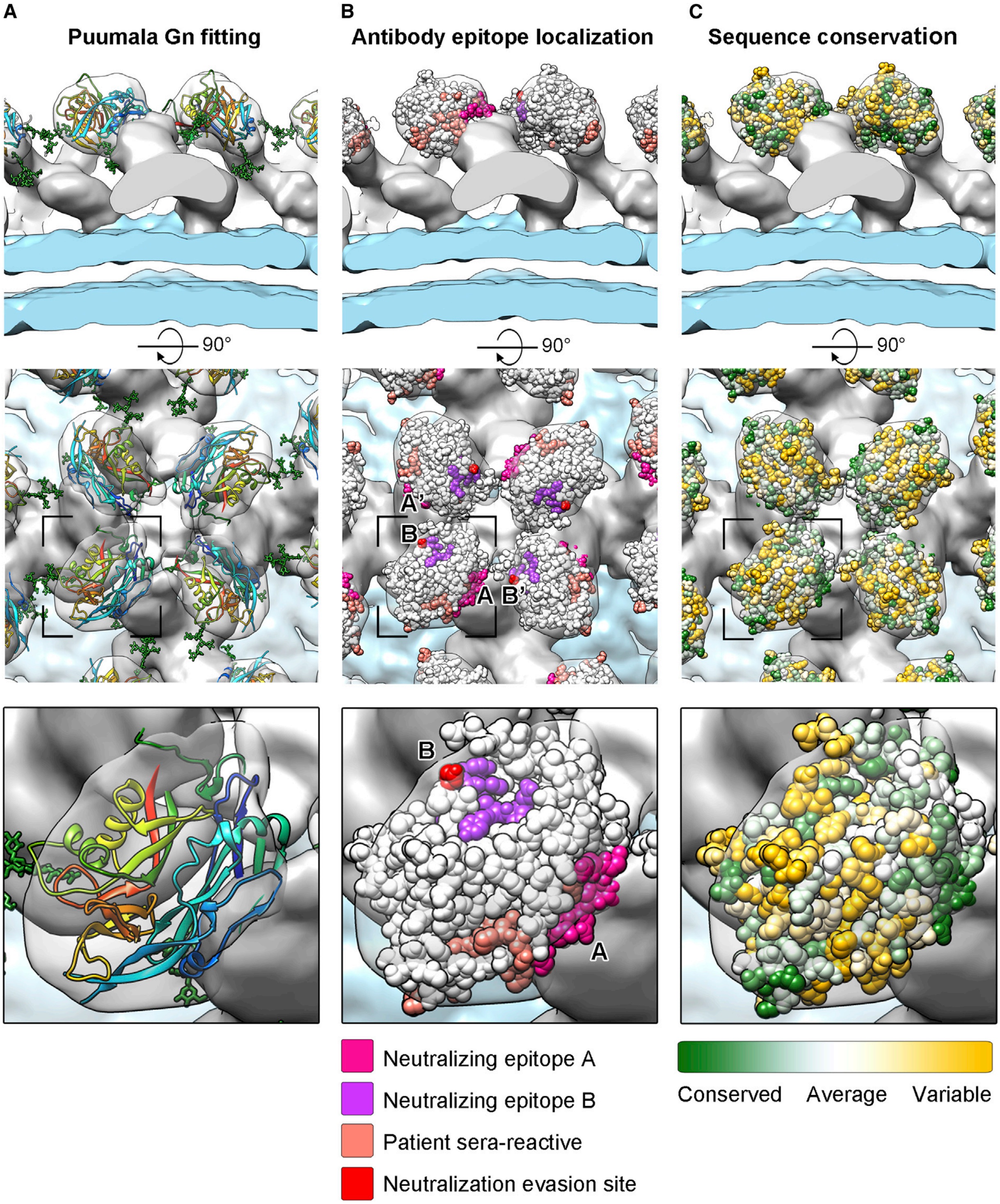

Figure 3. Mapping Functional Residues onto PUUV Gn Surface

(A) PUUV Gn fitted into the TULV reconstruction, as in Figure 2, with zoom panel shown (bottom).

(B) Mapping the antigenic surface of PUUV Gn. Predicted mAb 5A2 neutralizing epitopes are colored magenta and purple (A/A′ and B/B′, respectively). Patient sera-reactive epitopes are colored salmon. The antibody neutralization evasion site (D272V) is colored red.

(C) Mapping sequence conservation onto PUUV Gn. Well-conserved (green), average (white), and variable (yellow) regions are shown. The conservation analysis was performed with Consurf (Ashkenazy et al., 2010) using the hantaviral sequences listed in the Figure S2 legend.

See also Figures S2–S5.

Cryo-ET, Sub-tomogram Averaging, and Gn Fitting

A 3-μl aliquot of purified TULV and 3 μl of colloidal 10-nm gold (Aurion) were applied on a plasma-cleaned EM grid (C-flat; Protochips). Grids were blotted for 3 s followed by plunge-freezing into a mixture of liquid ethane (37%) and propane (63%) (Tivol et al., 2008).

Data were collected using a Tecnai F30 "Polara" transmission electron microscope (FEI) operated at 300 KV and at liquid nitrogen temperature. SerialEM (Mastronarde, 2005) was used to acquire tomographic tilt series on a direct electron detector (K2 Summit; Gatan) mounted behind an energy filter (QIF Quantum LS; Gatan) operated at zero-energy-loss mode (slit width, 20 eV). Movies consisting of eight frames (total exposure 1.6 s) were acquired at each tilt in electron-counting superresolution mode at a calibrated magnification of ×37,037, corresponding to a pixel size of 0.675 Å. Defocus values used were from 2.0 to 3.8 μm.

To correct for beam induced motion, frames at each tilt were aligned and averaged, and 2× binning was applied (Li et al., 2013). 3D tomograms were reconstructed using IMOD (Kremer et al., 1996). The gold beads were used as fiducial markers to align the images and were computationally removed prior to reconstruction. Contrast transfer function parameters were estimated and images corrected by phase flipping (Xiong et al., 2009). Further 2× binning was applied, resulting in the final pixel size of 2.7 Å.

Sub-tomogram averaging was carried out in Dynamo (Castaño-Díez et al., 2012) using a previously determined structure of the TULV spike (EMDB: 1704) as an initial template and following an iterative gold-standard alignment strategy (Huiskonen et al., 2014; Li et al., 2016). To reduce template bias, the initial template was filtered to 43-Å resolution (see Supplemental Experimental Procedures). The resolution of the final averaged density map was estimated by FSC using a criterion of 0.143.

The fitting of PUUV Gn into the TULV EM density map was performed with Segger (Pintilie et al., 2010) in Chimera (Pettersen et al., 2004) and is further described in Supplemental Experimental Procedures.

Evolutionary Conservation of Amino Acid Residues

For dN/dS analysis, a dataset of 25 PUUV glycoprotein sequences were collated from GenBank. A multiple sequence alignment was generated using MUSCLE (Edgar, 2004) and average dN/dS values for Gn and Gc were estimated using the SLAC model implemented in the HYPHY package (Kosakovsky Pond and Frost, 2005; Pond et al., 2005).

Evolutionary conservation of amino acid residues was mapped onto the PUUV Gn structure using ConSurf (Ashkenazy et al., 2010) with a multiple sequence alignment of 39 hantavirus Gn sequences (generated using MUSCLE; Edgar, 2004) and a maximum likelihood phylogenetic tree (LG + G + I model; Le and Gascuel, 2008) generated with MEGA6 (Tamura et al., 2013). GenBank accession numbers are listed in the legend to Figure S2. Conservation scores were calculated using an empirical Bayesian algorithm (Mayrose et al., 2004). An LG evolutionary substitution model (Le and Gascuel, 2008) was applied.

ACCESSION NUMBERS

The accession number for the atomic coordinates and structure factors of PUUV Gn reported in this paper is PDB: 5FXU. The accession numbers for the EM structure of the TULV Gn-Gc complex and the fitted PUUV Gn coordinates are EMDB: EMD-3364 and PDB: 4FYN, respectively.

SUPPLEMENTAL INFORMATION

Supplemental Information includes Supplemental Experimental Procedures and five figures and can be found with this article online at http://dx.doi.org/10.1016/j.celrep.2016.03.082.

AUTHOR CONTRIBUTIONS

All authors designed and performed the experiments, analyzed the data, and wrote the manuscript.

ACKNOWLEDGMENTS

We are grateful to Weixian Lu for help with tissue culture, Alistair Siebert for EM support, and the staff of beamlines I03 and I04 at DLS for assistance. We acknowledge the use of the University of Oxford Advanced Research Computing (ARC) facility (http://zenodo.org/record/22558). The Oxford Particle Imaging Centre was founded by a Wellcome Trust JIF award (060208/Z/00/Z) and is supported by a WT equipment grant (093305/Z/10/Z). The work was funded by the MRC (MR/J007897/1 to T.A.B and J.T.H, MR/N00065X/1 to K.H., and MR/L009528/1 to T.A.B.), the European Commission (658363 to A.Z.), and the European Research Council (ERC) under the European Union's Horizon 2020 research and innovation program (649053 to J.T.H.). O.G.P. was supported by the ERC under the European Commission Seventh Framework Programme (FP7/2007-2013)/ERC grant agreement 614725-PATHPHYLODYN. The Wellcome Trust Centre for Human Genetics is supported by Wellcome Trust Centre grant 090532/Z/09/Z. This paper is dedicated to the memory of Richard M. Elliott.

Received: September 23, 2015
Revised: January 29, 2016
Accepted: March 22, 2016
Published: April 21, 2016

REFERENCES

Acuña, R., Bignon, E.A., Mancini, R., Lozach, P.Y., and Tischler, N.D. (2015). Acidification triggers Andes hantavirus membrane fusion and rearrangement of Gc into a stable post-fusion homotrimer. J. Gen. Virol. *96*, 3192–3197.

Aricescu, A.R., Lu, W., and Jones, E.Y. (2006). A time- and cost-efficient system for high-level protein production in mammalian cells. Acta Crystallogr. D Biol. Crystallogr. *62*, 1243–1250.

Arikawa, J., Schmaljohn, A.L., Dalrymple, J.M., and Schmaljohn, C.S. (1989). Characterization of Hantaan virus envelope glycoprotein antigenic determinants defined by monoclonal antibodies. J. Gen. Virol. *70*, 615–624.

Ashkenazy, H., Erez, E., Martz, E., Pupko, T., and Ben-Tal, N. (2010). ConSurf 2010: calculating evolutionary conservation in sequence and structure of proteins and nucleic acids. Nucleic Acids Res. *38*, W529–W533.

Bammes, B.E., Rochat, R.H., Jakana, J., Chen, D.H., and Chiu, W. (2012). Direct electron detection yields cryo-EM reconstructions at resolutions beyond 3/4 Nyquist frequency. J. Struct. Biol. *177*, 589–601.

Battisti, A.J., Chu, Y.K., Chipman, P.R., Kaufmann, B., Jonsson, C.B., and Rossmann, M.G. (2011). Structural studies of Hantaan virus. J. Virol. *85*, 835–841.

Bowden, T.A., Crispin, M., Graham, S.C., Harvey, D.J., Grimes, J.M., Jones, E.Y., and Stuart, D.I. (2009). Unusual molecular architecture of the machupo virus attachment glycoprotein. J. Virol. *83*, 8259–8265.

Bowden, T.A., Crispin, M., Harvey, D.J., Jones, E.Y., and Stuart, D.I. (2010). Dimeric architecture of the Hendra virus attachment glycoprotein: evidence for a conserved mode of assembly. J. Virol. *84*, 6208–6217.

Bowden, T.A., Bitto, D., McLees, A., Yeromonahos, C., Elliott, R.M., and Huiskonen, J.T. (2013). Orthobunyavirus ultrastructure and the curious tripodal glycoprotein spike. PLoS Pathog. *9*, e1003374.

Buranda, T., Wu, Y., Perez, D., Jett, S.D., BonduHawkins, V., Ye, C., Edwards, B., Hall, P., Larson, R.S., Lopez, G.P., et al. (2010). Recognition of decay accelerating factor and alpha(v)beta(3) by inactivated hantaviruses: Toward the development of high-throughput screening flow cytometry assays. Anal. Biochem. *402*, 151–160.

Castaño-Díez, D., Kudryashev, M., Arheit, M., and Stahlberg, H. (2012). Dynamo: a flexible, user-friendly development tool for subtomogram averaging of cryo-EM data in high-performance computing environments. J. Struct. Biol. *178*, 139–151.

Choi, Y., Kwon, Y.C., Kim, S.I., Park, J.M., Lee, K.H., and Ahn, B.Y. (2008). A hantavirus causing hemorrhagic fever with renal syndrome requires gC1qR/p32 for efficient cell binding and infection. Virology *381*, 178–183.

Chothia, C., and Lesk, A.M. (1986). The relation between the divergence of sequence and structure in proteins. EMBO J. *5*, 823–826.

Crispin, M., Harvey, D.J., Bitto, D., Halldorsson, S., Bonomelli, C., Edgeworth, M., Scrivens, J.H., Huiskonen, J.T., and Bowden, T.A. (2014). Uukuniemi Phlebovirus assembly and secretion leave a functional imprint on the virion glycome. J. Virol. *88*, 10244–10251.

Dessau, M., and Modis, Y. (2013). Crystal structure of glycoprotein C from Rift Valley fever virus. Proc. Natl. Acad. Sci. USA *110*, 1696–1701.

Edgar, R.C. (2004). MUSCLE: multiple sequence alignment with high accuracy and high throughput. Nucleic Acids Res. *32*, 1792–1797.

Estrada, D.F., Boudreaux, D.M., Zhong, D., St Jeor, S.C., and De Guzman, R.N. (2009). The Hantavirus Glycoprotein G1 Tail Contains Dual CCHC-type Classical Zinc Fingers. J. Biol. Chem. *284*, 8654–8660.

Estrada, D.F., Conner, M., Jeor, S.C., and Guzman, R.N. (2011). The structure of the hantavirus zinc finger domain is conserved and represents the only natively folded region of the Gn cytoplasmic tail. Front. Microbiol. *2*, 251.

Gavrilovskaya, I.N., Shepley, M., Shaw, R., Ginsberg, M.H., and Mackow, E.R. (1998). beta3 Integrins mediate the cellular entry of hantaviruses that cause respiratory failure. Proc. Natl. Acad. Sci. USA *95*, 7074–7079.

Heiskanen, T., Lundkvist, A., Soliymani, R., Koivunen, E., Vaheri, A., and Lankinen, H. (1999). Phage-displayed peptides mimicking the discontinuous neutralization sites of puumala Hantavirus envelope glycoproteins. Virology *262*, 321–332.

Heiskanen, T., Li, X.D., Hepojoki, J., Gustafsson, E., Lundkvist, A., Vaheri, A., and Lankinen, H. (2003). Improvement of binding of Puumala virus neutralization site resembling peptide with a second-generation phage library. Protein Eng. *16*, 443–450.

Hepojoki, J., Strandin, T., Vaheri, A., and Lankinen, H. (2010). Interactions and oligomerization of hantavirus glycoproteins. J. Virol. *84*, 227–242.

Hörling, J., and Lundkvist, A. (1997). Single amino acid substitutions in Puumala virus envelope glycoproteins G1 and G2 eliminate important neutralization epitopes. Virus Res. *48*, 89–100.

Huiskonen, J.T., Hepojoki, J., Laurinmäki, P., Vaheri, A., Lankinen, H., Butcher, S.J., and Grünewald, K. (2010). Electron cryotomography of Tula hantavirus suggests a unique assembly paradigm for enveloped viruses. J. Virol. *84*, 4889–4897.

Huiskonen, J.T., Parsy, M.L., Li, S., Bitto, D., Renner, M., and Bowden, T.A. (2014). Averaging of viral envelope glycoprotein spikes from electron cryotomography reconstructions using Jsubtomo. J. Vis. Exp. *92*, e51714.

Jenison, S., Yamada, T., Morris, C., Anderson, B., Torrez-Martinez, N., Keller, N., and Hjelle, B. (1994). Characterization of human antibody responses to four corners hantavirus infections among patients with hantavirus pulmonary syndrome. J. Virol. *68*, 3000–3006.

Jin, M., Park, J., Lee, S., Park, B., Shin, J., Song, K.J., Ahn, T.I., Hwang, S.Y., Ahn, B.Y., and Ahn, K. (2002). Hantaan virus enters cells by clathrin-dependent receptor-mediated endocytosis. Virology *294*, 60–69.

Johansson, P., Olsson, M., Lindgren, L., Ahlm, C., Elgh, F., Holmström, A., and Bucht, G. (2004). Complete gene sequence of a human Puumala hantavirus isolate, Puumala Umeå/hu: sequence comparison and characterisation of encoded gene products. Virus Res. *105*, 147–155.

Jonsson, C.B., Figueiredo, L.T., and Vapalahti, O. (2010). A global perspective on hantavirus ecology, epidemiology, and disease. Clin. Microbiol. Rev. *23*, 412–441.

Julien, J.P., Lee, J.H., Cupo, A., Murin, C.D., Derking, R., Hoffenberg, S., Caulfield, M.J., King, C.R., Marozsan, A.J., Klasse, P.J., et al. (2013). Asymmetric recognition of the HIV-1 trimer by broadly neutralizing antibody PG9. Proc. Natl. Acad. Sci. USA *110*, 4351–4356.

Koch, J., Liang, M., Queitsch, I., Kraus, A.A., and Bautz, E.K. (2003). Human recombinant neutralizing antibodies against hantaan virus G2 protein. Virology *308*, 64–73.

Kosakovsky Pond, S.L., and Frost, S.D. (2005). Not so different after all: a comparison of methods for detecting amino acid sites under selection. Mol. Biol. Evol. *22*, 1208–1222.

Kremer, J.R., Mastronarde, D.N., and McIntosh, J.R. (1996). Computer visualization of three-dimensional image data using IMOD. J. Struct. Biol. *116*, 71–76.

Krogh, A., Larsson, B., von Heijne, G., and Sonnhammer, E.L. (2001). Predicting transmembrane protein topology with a hidden Markov model: application to complete genomes. J. Mol. Biol. *305*, 567–580.

Le, S.Q., and Gascuel, O. (2008). An improved general amino acid replacement matrix. Mol. Biol. Evol. *25*, 1307–1320.

Lednicky, J.A. (2003). Hantaviruses. a short review. Arch. Pathol. Lab. Med. *127*, 30–35.

Lee, H.W., and Johnson, K.M. (1982). Laboratory-acquired infections with Hantaan virus, the etiologic agent of Korean hemorrhagic fever. J. Infect. Dis. *146*, 645–651.

Li, L., Jose, J., Xiang, Y., Kuhn, R.J., and Rossmann, M.G. (2010). Structural changes of envelope proteins during alphavirus fusion. Nature *468*, 705–708.

Li, X., Mooney, P., Zheng, S., Booth, C.R., Braunfeld, M.B., Gubbens, S., Agard, D.A., and Cheng, Y. (2013). Electron counting and beam-induced motion correction enable near-atomic-resolution single-particle cryo-EM. Nat. Methods *10*, 584–590.

Li, S., Sun, Z., Pryce, R., Parsy, M.L., Fehling, S.K., Schlie, K., Siebert, C.A., Garten, W., Bowden, T.A., Strecker, T., and Huiskonen, J.T. (2016). Acidic pH-induced conformations and LAMP1 binding of the Lassa virus glycoprotein spike. PLoS Pathog. *12*, e1005418.

Liang, M., Mahler, M., Koch, J., Ji, Y., Li, D., Schmaljohn, C., and Bautz, E.K. (2003). Generation of an HFRS patient-derived neutralizing recombinant antibody to Hantaan virus G1 protein and definition of the neutralizing domain. J. Med. Virol. *69*, 99–107.

Löber, C., Anheier, B., Lindow, S., Klenk, H.D., and Feldmann, H. (2001). The Hantaan virus glycoprotein precursor is cleaved at the conserved pentapeptide WAASA. Virology *289*, 224–229.

Lundkvist, A., and Niklasson, B. (1992). Bank vole monoclonal antibodies against Puumala virus envelope glycoproteins: identification of epitopes involved in neutralization. Arch. Virol. *126*, 93–105.

Lundkvist, A., Hörling, J., Athlin, L., Rosén, A., and Niklasson, B. (1993). Neutralizing human monoclonal antibodies against Puumala virus, causative agent of nephropathia epidemica: a novel method using antigen-coated magnetic beads for specific B cell isolation. J. Gen. Virol. *74*, 1303–1310.

Mastronarde, D.N. (2005). Automated electron microscope tomography using robust prediction of specimen movements. J. Struct. Biol. *152*, 36–51.

Mayrose, I., Graur, D., Ben-Tal, N., and Pupko, T. (2004). Comparison of site-specific rate-inference methods for protein sequences: empirical Bayesian methods are superior. Mol. Biol. Evol. *21*, 1781–1791.

Nuzum, E.O., Rossi, C.A., Stephenson, E.H., and LeDuc, J.W. (1988). Aerosol transmission of Hantaan and related viruses to laboratory rats. Am. J. Trop. Med. Hyg. *38*, 636–640.

Petersen, T.N., Brunak, S., von Heijne, G., and Nielsen, H. (2011). SignalP 4.0: discriminating signal peptides from transmembrane regions. Nat. Methods *8*, 785–786.

Pettersen, E.F., Goddard, T.D., Huang, C.C., Couch, G.S., Greenblatt, D.M., Meng, E.C., and Ferrin, T.E. (2004). UCSF Chimera–a visualization system for exploratory research and analysis. J. Comput. Chem. *25*, 1605–1612.

Pintilie, G.D., Zhang, J., Goddard, T.D., Chiu, W., and Gossard, D.C. (2010). Quantitative analysis of cryo-EM density map segmentation by watershed and scale-space filtering, and fitting of structures by alignment to regions. J. Struct. Biol. *170*, 427–438.

Pond, S.L., Frost, S.D., and Muse, S.V. (2005). HyPhy: hypothesis testing using phylogenies. Bioinformatics *21*, 676–679.

Raymond, T., Gorbunova, E., Gavrilovskaya, I.N., and Mackow, E.R. (2005). Pathogenic hantaviruses bind plexin-semaphorin-integrin domains present at the apex of inactive, bent alphavbeta3 integrin conformers. Proc. Natl. Acad. Sci. USA *102*, 1163–1168.

Ren, J., Wen, L., Gao, X., Jin, C., Xue, Y., and Yao, X. (2009). DOG 1.0: illustrator of protein domain structures. Cell Res. *19*, 271–273.

Schmaljohn, C.S., Schmaljohn, A.L., and Dalrymple, J.M. (1987). Hantaan virus M RNA: coding strategy, nucleotide sequence, and gene order. Virology *157*, 31–39.

Schmaljohn, C.S., Chu, Y.K., Schmaljohn, A.L., and Dalrymple, J.M. (1990). Antigenic subunits of Hantaan virus expressed by baculovirus and vaccinia virus recombinants. J. Virol. *64*, 3162–3170.

Shi, X., and Elliott, R.M. (2004). Analysis of N-linked glycosylation of hantaan virus glycoproteins and the role of oligosaccharide side chains in protein folding and intracellular trafficking. J. Virol. *78*, 5414–5422.

Spiropoulou, C.F., Goldsmith, C.S., Shoemaker, T.R., Peters, C.J., and Compans, R.W. (2003). Sin Nombre virus glycoprotein trafficking. Virology *308*, 48–63.

Tamura, K., Stecher, G., Peterson, D., Filipski, A., and Kumar, S. (2013). MEGA6: Molecular Evolutionary Genetics Analysis version 6.0. Mol. Biol. Evol. *30*, 2725–2729.

Tischler, N.D., Gonzalez, A., Perez-Acle, T., Rosemblatt, M., and Valenzuela, P.D. (2005). Hantavirus Gc glycoprotein: evidence for a class II fusion protein. J. Gen. Virol. *86*, 2937–2947.

Tivol, W.F., Briegel, A., and Jensen, G.J. (2008). An improved cryogen for plunge freezing. Microsc. Microanal. *14*, 375–379.

Vaheri, A., Strandin, T., Hepojoki, J., Sironen, T., Henttonen, H., Mäkelä, S., and Mustonen, J. (2013). Uncovering the mysteries of hantavirus infections. Nat. Rev. Microbiol. *11*, 539–550.

Xiong, Q., Morphew, M.K., Schwartz, C.L., Hoenger, A.H., and Mastronarde, D.N. (2009). CTF determination and correction for low dose tomographic tilt series. J. Struct. Biol. *168*, 378–387.

Cell Reports, Volume *15*

Supplemental Information

A Molecular-Level Account

# of the Antigenic Hantaviral Surface

Sai Li, Ilona Rissanen, Antra Zeltina, Jussi Hepojoki, Jayna Raghwani, Karl Harlos, Oliver G. Pybus, Juha T. Huiskonen, and Thomas A. Bowden

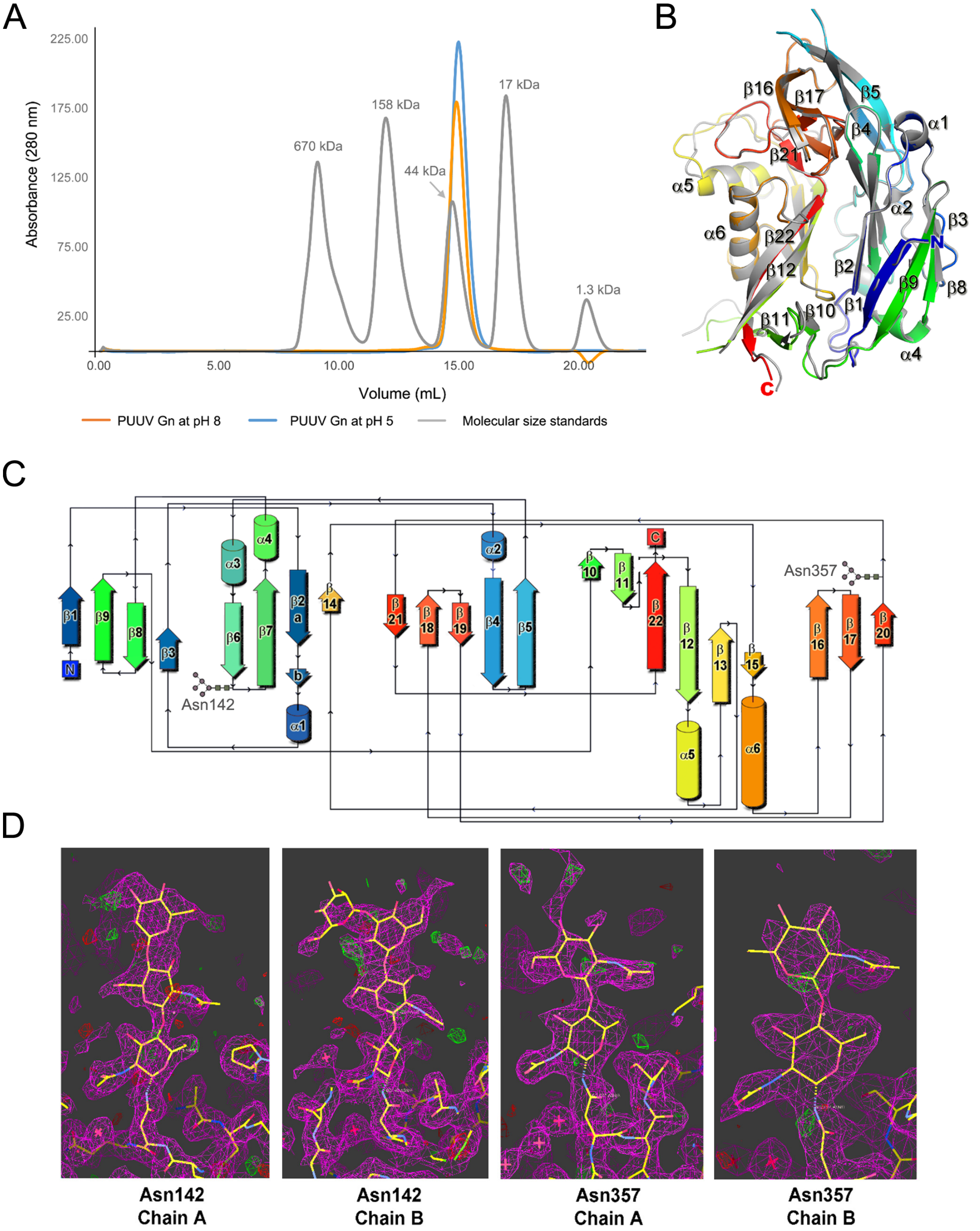

Figure S1. Structural and biochemical analysis of Puumala virus (PUUV) Gn, related to Figure 1 and Table 1. (A) Size exclusion chromatography analysis of PUUV Gn was run on a Superdex 200 10/30 column equilibrated in 150 mM NaCl and 10 mM Tris, pH 8.0 (orange curve), overlaid with a chromatogram of gel filtration standards (grey curve, Bio-Rad #151-1901) run in similar conditions. Gel filtration standard peaks correspond to thyroglobulin (670 kDa), γ-globulin (158 kDa), ovalbumin (44 kDa), myoglobin (17 kDa) and vitamin B12 (1.35 kDa). Size exclusion chromatography of PUUV Gn was also performed in 150 mM NaCl and 10 mM citrate, pH 5.0 (blue curve) and reveals that PUUV Gn remains as a putative monomeric species at pH 5.0. (B) Structural overlay of the two PUUV Gn chains observed in the asymmetric unit. Shown in cartoon

representation with one molecule colored gray and the other as a rainbow ramped from blue (N-terminus) to red (C-terminus). (C) Topology diagram of PUUV Gn ectodomain (generated with PDBsum (Laskowski, 2009)). Secondary structure elements are numbered starting from the N-terminus and shown with an arrow (β-strand) or cylinder (α-helices and the 3_{10}-type helix, α2) and are colored as in panel *B*. (D) Electron density at N-linked glycosylation sequons in the PUUV Gn crystal structure. Maximum likelihood-weighted 2*Fo*−*Fc* (pink) and *Fo*−*Fc* (green and red) electron density is shown at each N-linked glycosylation sequon. Well-ordered N-linked glycans were observed at Asn142 and Asn357 for both molecules (chains A and B) observed in the asymmetric unit of the crystal.

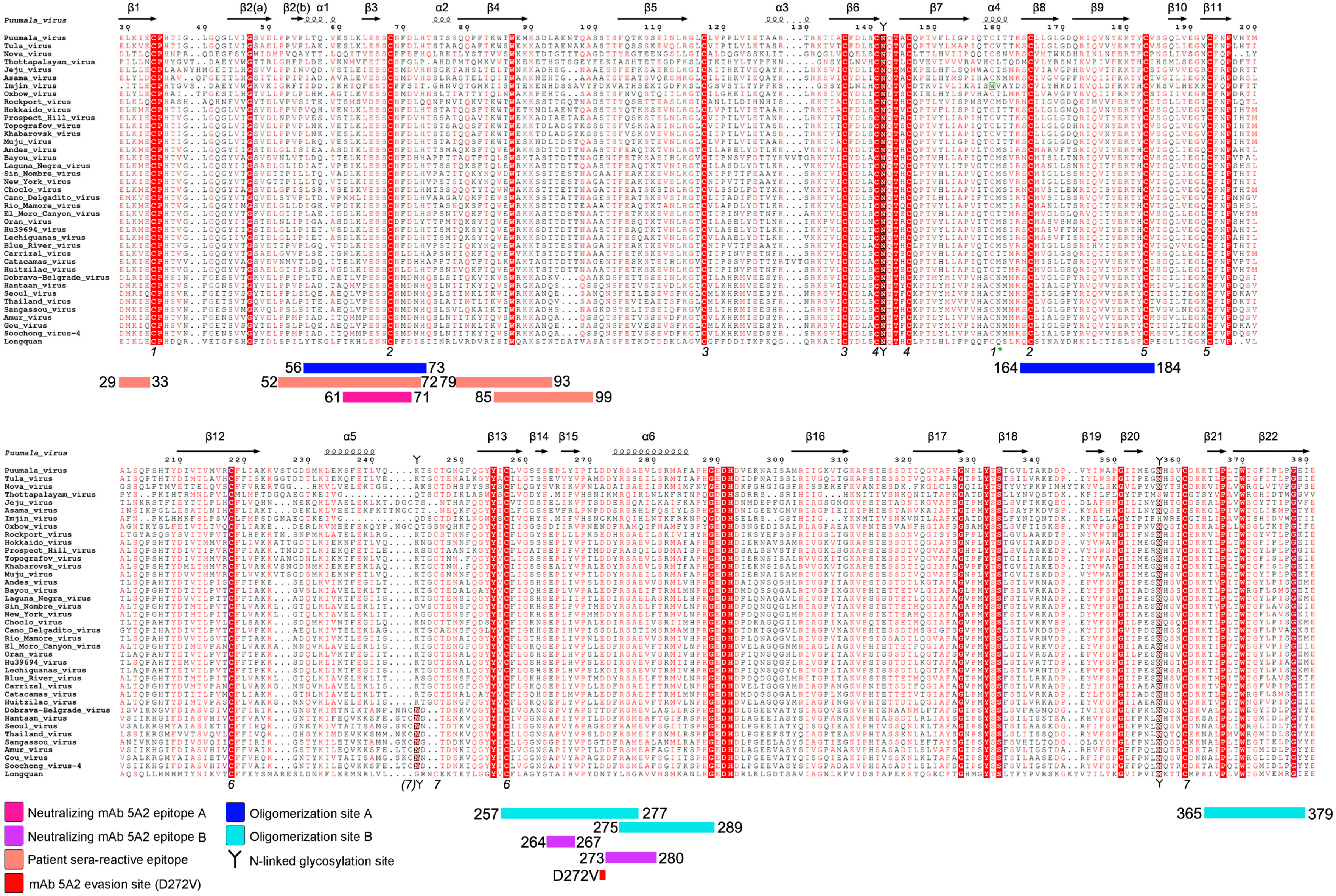
Neutralizing mAb 5A2 epitope A
Neutralizing mAb 5A2 epitope B
Patient sera-reactive epitope
mAb 5A2 evasion site (D272V)
Oligomerization site A
Oligomerization site B
N-linked glycosylation site

Figure S2. Sequence alignment and mapping of functional residues included in the crystallized PUUV Gn with other hantaviral Gn glycoproteins, related to Figure 1 and 3. The alignment was prepared with ClustalW (Chenna et al., 2003) and plotted with ESPRIPT (Gouet et al., 1999). Residues which are highlighted in red are fully conserved, residues which are colored red are partially conserved, and residues which are colored black are not conserved. Amino acids corresponding to predicted N-linked glycosylation sites are outlined in black and marked with black trees. Secondary structure from the PUUV crystal structure was mapped above the alignment, where helices, including the 3_{10}-type helix, α2, are shown as spirals and β-strands are shown as arrows. Cysteines participating in disulfide bonds are numbered, in italics, underneath the alignment, and the single instance (Imjin virus) where the cysteine pattern is not conserved is annotated with a green box. Cysteines likely participating in disulfide bond seven occupy slightly different positions in the sequences of different viruses, and are thus denoted by (7) and 7. The epitopes illustrated in main Figure 3 are presented as bars below the alignment as follows: predicted neutralizing and patient sera-reactive epitopes (Heiskanen et al., 1999) are shown in magenta and purple (mAb 5A2 epitopes A and B, respectively) and salmon (patient sera-reactive epitopes) and the mAb 5A2 neutralization evasion site (D272V) (Horling and Lundkvist, 1997) is shown in red. In the context of our preferred Gn_{ecto} fitting into the Tula reconstruction (main Figure 3), these antigenic regions are solvent exposed. Interestingly, the PUUV antigenic epitopes around the mAb 5A2 epitope A also overlap with the previously identified immunodominant epitope in Sin Nombre virus (SNV residues 59-89) (Jenison et al., 1994).The two sets of oligomerization sites identified on the Gn surface (Hepojoki et al., 2010) are annotated dark blue (site A) and cyan (site B). Residues 164-184 and 365-379 are buried within the putative oligomerization interface, while residues 56-73 (site A) and residues 257-289 (site B) exhibit a greater degree of solvent accessibility. GenBank accession numbers for the used sequences are as follows: Puumala virus CAB43026.1; Hokkaido virus BAM13711.1; Muju virus AGE45110.1; Khabarovsk virus AIL25323.1; Topografov virus CAB42098.1; Tula virus NP_942586.1; Prospect Hill virus CAA38922.1, Huitzilac virus BAK08526.1; El Moro Canyon virus AAA87198.1; Blue River virus AAC03793.1; Sin Nombre virus AFV71282.1; Bayou virus AAA61690.1; Cano Delgadito virus ABB88646.1; Andes virus AAO86638.1; Oran virus AAB87910.1; Laguna Negra virus AAB87603.1; Rio Mamore virus ACU46022.1; Choclo virus ABB90558.1; Hantaan virus BAA05012.1; Amur virus AGE13901.1; Seoul virus ABW05099.1; Thailand virus AAA16239.1; Dobrava-Belgrade virus ADP21266.1; Sangassou virus AEZ02947.1; Asama virus ACI28506.1; Oxbow virus ACT68338.1; Jeju virus AEX56232.1; Nova virus AKE37996.1; Thottapalayam virus AIF28800.1; Imjin virus AIF28793.1; Rockport virus AEA11485.1; Longquan virus AGI62348.1, Soochong virus-4 AAY56327.1; Gou virus AGC97139.1; Hu39694 virus AAB87909.1; Lechiguanas virus AAB87908.1; Catacamas virus ABA39272.1; New York virus AAC54561.1; Carrizal virus BAK08523.1.

#	Virus	1	2	3	4	5	6	7	8	9	10	11	12	13	14	15	16	17	18	19	20	21	22	23	24	25	26	27	28	29	30	31	32	33	34	35	36	37	38	39
1	Thottapalayam virus																																							
2	Imjin_virus	70.2																																						
3	Longquan virus	38.9	39.7																																					
4	Nova virus	42.5	41.9	44.9																																				
5	Rockport virus	43.1	41.9	41.7	48.9																																			
6	Prospect Hill virus	42.7	42.5	41.6	47.3	61.6																																		
7	Tula virus	42.7	44.1	40.3	47.4	63.3	79.7																																	
8	Puumala virus	42.6	42.5	40.9	46.4	63.2	75.6	78.6																																
9	Hokkaido virus	43.0	42.5	40.9	46.0	62.2	77.0	80.0	91.3																															
10	Muju virus	42.6	42.4	40.6	46.5	63.1	76.4	79.4	89.4	92.4																														
11	Topografov virus	43.5	42.9	41.1	46.9	62.3	77.2	81.1	82.6	83.9	84.6																													
12	Khabarovsk virus	43.2	42.6	41.9	46.5	62.4	78.4	81.3	82.9	84.2	85.4	90.4																												
13	Cano Delgadito virus	43.1	42.5	41.0	48.3	62.2	64.5	66.0	64.5	64.9	65.2	66.0	65.7																											
14	Choclo virus	43.7	43.7	40.2	46.8	61.4	65.8	66.4	64.4	65.1	65.1	65.5	66.1	76.4																										
15	Andes virus	43.4	44.9	40.6	46.4	62.5	66.5	67.7	66.1	66.2	66.4	67.7	66.8	73.8	82.4																									
16	Oran virus	43.0	44.2	40.6	46.8	61.9	66.3	67.9	65.6	66.0	66.5	67.3	66.8	73.7	82.3	91.9																								
17	Hu39694 virus	43.2	44.3	40.2	46.7	61.7	66.6	68.0	65.8	66.2	66.7	67.8	67.4	73.5	81.9	92.0	94.1																							
18	Lechiguanas virus	43.5	44.8	40.8	47.2	62.3	66.7	68.3	65.8	66.3	66.7	67.8	67.2	73.7	83.0	93.0	95.4	95.5																						
19	Laguna Negra virus	43.2	44.0	39.8	46.1	61.1	65.9	67.4	65.4	65.9	66.9	67.1	66.9	73.9	81.6	86.4	86.3	86.3	86.6																					
20	Rio Mamore virus	43.7	44.9	41.1	46.5	60.9	66.6	68.2	66.2	66.5	66.8	67.8	67.3	74.7	83.1	87.4	88.3	88.0	88.9	91.2																				
21	Bayou virus	42.8	43.1	40.1	46.2	61.6	66.1	68.2	64.6	64.6	65.6	67.5	67.4	75.8	77.4	75.2	75.7	75.5	75.6	75.6	75.8																			
22	Catacamas virus	42.7	43.2	39.6	46.1	61.7	65.5	68.3	64.0	64.2	65.3	67.0	67.1	75.3	76.8	74.9	75.2	75.0	75.5	75.5	75.0	93.6																		
23	Blue River virus	42.3	43.0	39.9	46.7	61.9	67.5	69.1	67.7	67.8	68.2	68.0	68.5	76.5	77.3	76.9	77.0	76.9	77.2	76.2	76.4	79.8	79.5																	
24	Sin Nombre virus	42.7	43.1	41.0	46.7	62.6	67.3	69.1	67.7	67.3	67.5	67.4	68.1	77.2	78.0	77.5	77.5	77.5	78.1	76.6	77.1	80.5	80.3	93.8																
25	New York virus	42.4	43.3	40.5	46.7	62.0	67.5	69.2	67.7	67.4	67.8	67.7	68.0	76.1	76.9	76.7	77.2	77.4	77.6	76.2	76.4	79.5	79.7	93.8	95.5															
26	El Moro Canyon virus	43.5	42.9	40.4	46.5	61.3	66.5	68.5	66.3	66.7	67.0	67.8	67.5	73.6	74.8	73.3	72.9	73.3	73.7	74.2	74.4	76.6	76.2	78.8	79.5	78.8														
27	Carrizal virus	43.2	43.0	40.5	47.3	62.8	67.4	68.9	67.1	66.8	67.7	67.9	68.5	74.6	76.5	74.4	75.2	74.7	75.7	75.0	75.0	77.9	77.5	79.9	80.4	79.6	90.1													
28	Huitzilac virus	43.2	42.4	40.7	47.2	62.7	67.1	69.1	67.5	67.0	67.9	69.1	68.6	74.9	75.9	75.3	75.0	74.9	75.3	75.3	75.1	78.3	77.5	81.0	81.9	80.7	91.0	93.0												
29	Hantaan virus	43.5	43.9	39.5	45.5	53.9	54.3	55.8	54.7	55.2	55.5	55.4	55.8	55.7	54.4	55.0	55.1	55.3	55.8	54.8	55.5	55.4	55.7	55.7	55.1	54.7	56.5	56.5	56.9											
30	Amur virus	43.5	44.4	39.9	45.3	53.1	54.7	54.9	55.0	55.9	55.0	55.3	55.9	55.1	54.4	55.2	55.4	55.4	55.7	55.4	56.4	55.3	55.2	56.0	55.2	54.9	55.8	56.0	56.4	91.7										
31	Soochong virus-4	43.4	44.4	39.6	45.4	53.3	54.3	55.0	54.8	55.9	54.9	54.9	56.1	55.7	54.8	55.0	55.3	55.5	55.7	55.6	56.4	55.5	55.7	56.3	55.6	55.0	56.3	56.5	56.7	92.2	97.2									
32	Thailand virus	43.4	44.6	39.3	46.4	53.0	54.1	54.6	54.0	54.6	55.5	54.4	55.0	54.7	53.7	53.9	54.5	54.3	54.7	53.6	54.7	54.6	54.9	54.0	53.9	53.9	55.8	56.4	56.2	77.1	76.2	76.9								
33	Seoul virus	43.0	43.4	38.7	45.6	53.6	53.8	54.4	54.1	53.8	54.4	54.5	55.5	54.3	53.3	54.3	54.7	54.4	54.8	54.1	54.3	54.3	54.3	53.3	53.3	53.1	54.4	55.3	55.3	76.4	75.8	76.2	81.5							
34	Gou virus	43.2	43.5	39.0	45.3	53.2	53.8	54.2	54.1	54.2	54.3	54.7	55.7	54.5	53.2	54.3	55.0	54.5	54.9	54.2	54.6	54.0	53.7	53.3	53.2	53.1	54.8	55.6	55.4	76.9	76.2	76.8	82.2	96.7						
35	Dobrava-Belgrade virus	43.8	44.4	39.6	45.7	53.4	53.9	55.2	55.0	55.0	54.6	54.6	55.3	54.9	54.5	54.7	54.4	54.7	55.1	54.4	54.9	55.4	55.2	55.0	55.2	54.7	55.2	55.7	56.0	77.0	76.4	76.7	76.3	76.6	76.4					
36	Sangassou virus	43.7	43.8	39.4	46.0	53.9	53.9	54.4	54.3	55.0	54.4	54.5	54.5	53.9	53.5	54.2	54.2	54.2	54.6	54.2	55.0	55.3	55.0	54.1	53.6	53.8	54.3	54.6	55.1	76.2	76.0	76.3	77.3	77.7	77.9	80.5				
37	Jeju virus	41.9	43.3	39.9	45.3	52.0	52.4	51.7	51.9	53.1	53.3	54.2	53.9	53.9	53.2	51.6	52.0	52.2	51.9	51.2	52.1	53.2	53.4	53.7	53.2	53.5	54.7	53.8	54.2	57.7	57.3	57.2	56.8	55.8	56.0	56.8	57.4			
38	Asama virus	41.9	43.3	39.8	46.3	51.5	51.7	52.6	52.4	52.6	51.9	52.3	52.4	54.7	53.8	53.7	53.8	53.6	53.6	53.8	53.7	53.2	53.8	54.7	54.1	54.5	54.5	54.1	54.9	59.6	60.0	60.5	59.9	59.1	59.6	59.9	58.6	64.5		
39	Oxbow virus	42.0	42.5	39.8	46.1	51.2	52.0	52.1	52.0	52.0	51.6	53.2	52.8	52.6	53.6	51.9	52.3	52.1	52.0	51.4	52.0	53.5	53.4	53.4	53.3	53.1	52.7	52.7	52.8	59.8	59.3	59.4	58.6	56.9	57.2	58.6	57.7	61.3	65.8	

Figure S3. Percent identity matrix of hantavirus glycoprotein precursor sequences, related to Figures 2 and 3. Colours indicate high (green) to low (red) amino acid identity values. Percent identity matrix and the underlying multiple sequence alignment were created by MUSCLE (Edgar, 2004). Names of the viruses borne by rodents of the family *Cricetidae* are shown in blue (subfamilies *Sigmodontinae* and *Neotominae*) and green (subfamily *Arvicolinae*). In gray, viruses that are carried by Old World mice and rats (family *Muridae*). Soricomorph- and bat-borne hantaviruses are shown in red and pink, respectively. The GenBank accession numbers used in the analysis are listed in Supplementary Figure Legend S2.

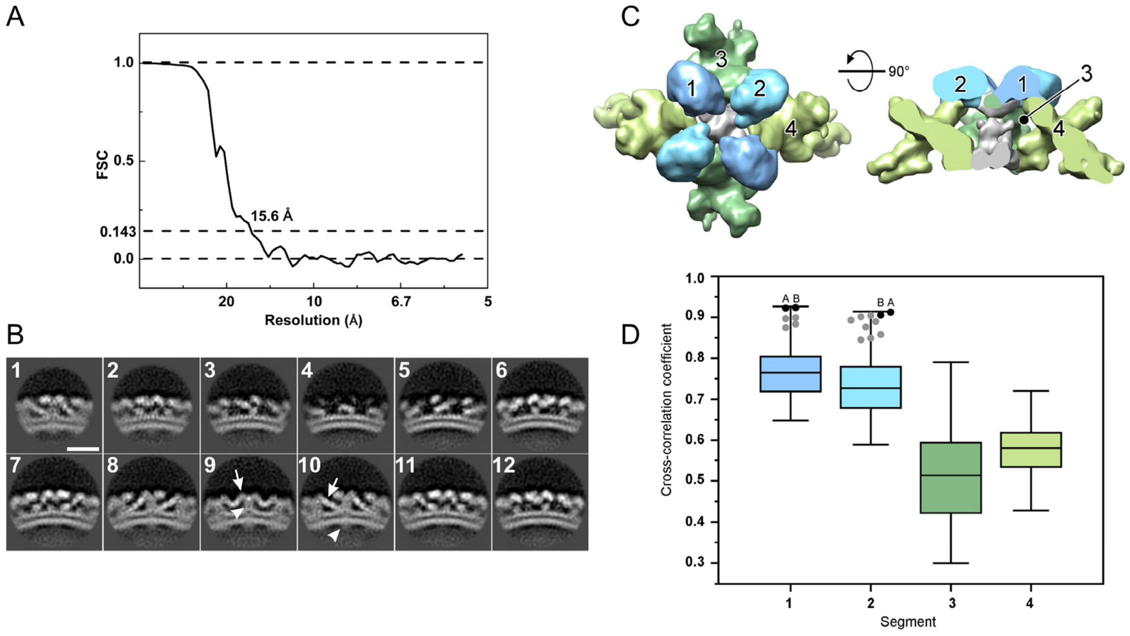

Figure S4. Analysis of the Tula virus (TULV) spike structure and fitting of PUUV Gn into the EM density, related to Figure 2, 3, and Table 2. (A) Fourier Shell Correlation (FSC) curve. To prevent overfitting during refinement, 'gold-standard' refinement was implemented by splitting the dataset into two halves and refining the two sets independently. At the end of each iteration, the FSC of the two averaged structures was computed, giving a resolution using criteria of 0.143, which was set as the low-pass for the next iteration. The final resolution of the structure was assessed by FSC analysis, giving 15.6 Å. (B) Slices through the TULV spike structure (1-12). Slices (1.35 nm thickness) through the volume from the side of the spike, nominal to the membrane. Putative regions corresponding to the Gc ectodomain (arrow, slice 10), Gc cytoplasmic domain (arrow head, slice 10), Gn membrane-distal domain (arrow, slice 9), and Gn membrane-proximal domain (arrow head, slice 9) are annotated. Scale bar, 14 nm. (C) Analysis of PUUV Gn ectodomain crystal structure fitting into TULV EM density. Density extracted from the TULV spike reconstruction is shown from the top (left) and side (right). Different density segments used in the fitting of the PUUV Gn ectodomain crystal structure are rendered in different colors and numbered. (D) A box-and-whisker plot showing the interquartile ranges of the cross-correlation coefficients between the crystal structure and four different segments (1–4) of the EM density. Cross-correlation values were calculated for 1000 evenly rotated fits (box plots). Segments 1 and 2, corresponding to the membrane-distal lobes of the spike tetramer, gave consistently higher cross-correlation values than segments 3 and 4, corresponding to the peripheral stalks of the spike. Two segments corresponding to the central stalk of the spike (rendered gray in panel *C*) were excluded from the analysis due to clashes between the fitted structure and its symmetry related copy. The top scoring optimized unique fits against segments 1 and 2 are denoted with gray and black circles. The two top fits (black circles labeled A and B) had very similar cross-correlation values (segment 1: A=0.922, B=0.924; segment 2: A=0.912, B=0.904) and correspond to the two alternative fitting solutions described in the maintext and in Figure S5.

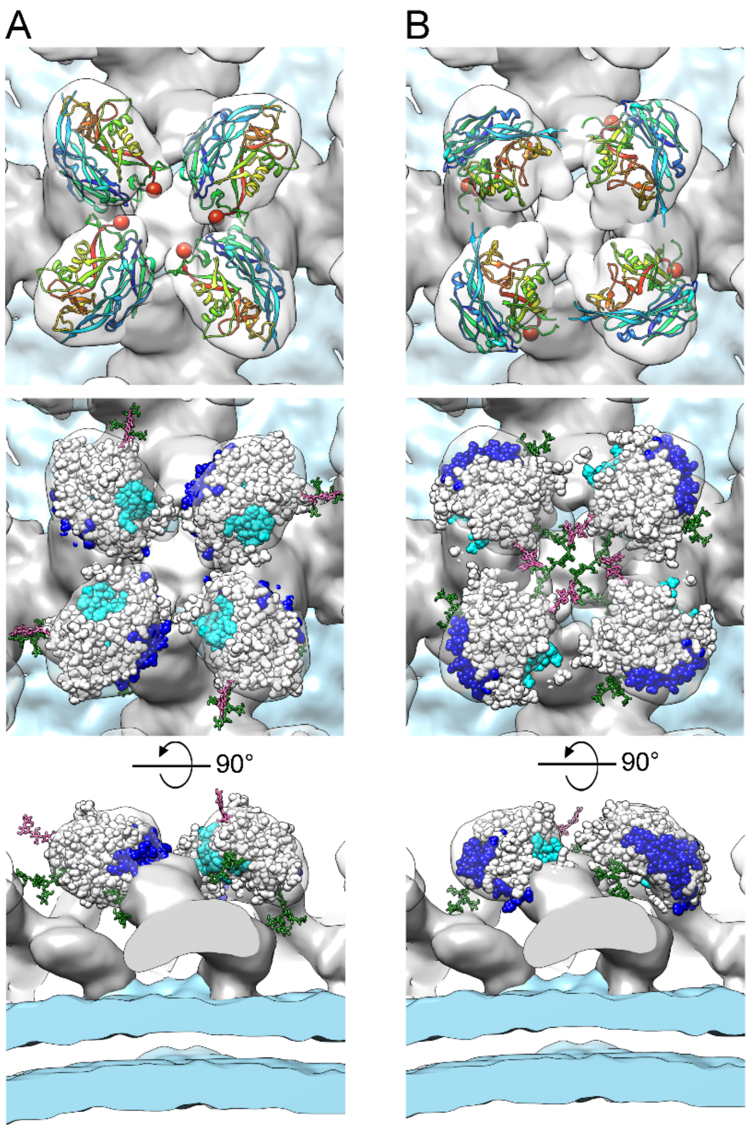

Figure S5. Fitting of PUUV Gn into the 16 Å TULV reconstruction yielded two types of possible solutions (panels *A* and *B*), related to Figure 2 and 3. Gn-Gc glycoprotein spikes are colored gray and the virion lipid bilayer envelope is colored light blue. The membrane-bound glycoprotein spike of TULV with the crystal structure of PUUV Gn fitted into the membrane distal lobes is shown. In the topmost panels, fitted PUUV Gn crystal structures are shown as ribbons colored as a rainbow ramped from blue (N-terminus) to red (C-terminus). Red spheres indicate the position of the C-termini for each fitted Gn. The two types of solutions generated by Segger (see **Supplementary Experimental Procedures**) prompted mapping of N-linked glycans and known oligomerization sites on the PUUV Gn surface as restraints to assess the validity of the fits. Sites of homotypic Gn oligomerisation (Hepojoki et al., 2010) are colored blue and cyan (as defined in **Figure S2**). As the hantaviral glycans presented on Gn and Gc are endoglycosidase H sensitive (Shi and Elliott, 2004), high mannose-type glycans (from PDB 2WAH (Crispin et al., 2009)) are modeled as green sticks at N-linked sequons (Asn142 and Asn357). Both of these sites were observed to be occupied in the crystal structure. An additional putative N-linked glycosylation site present within the Hantaan virus subgroup (defined in **Figure S2** and **S3**) was modeled onto PUUV Gn as pink sticks at the putative N-linked glycosylation sequon (Asn235 in

Hantaan virus and Lys243 in PUUV). As described in the main text, the following observations (*i*-*ii*) provide confidence to our selected fitting: (*i*) The chosen fit (panel *A*) localizes the Gn to the tetrameric, membrane-distal lobes of the spike such that C-terminal transmembrane anchor regions co-localize and point towards the virion envelope. In the rejected fit (panel *B*), the C-termini of the Gn are pointed outwards from the spike towards inter-glycoprotein spike solvent regions. (*ii*) For our selected PUUV Gn fitting (panel *A*), we observe that N-linked glycans on the Gn extend from the tetrameric glycoprotein spike surface into inter-spike solvent accessible regions. In contrast, these N-linked glycans sterically clash with neighboring glycans within the same spike in the alternative fit (panel *B*).

SUPPLEMENTARY EXPERIMENTAL PROCEDURES

Crystal structure determination and refinement

PUUV Gn was concentrated to 6.0 mg per mL in 150 mM NaCl and 10 mM Tris pH 8.0 and crystallized after 15 days using the vapour diffusion method (Walter et al., 2005) by mixing 100 nl protein plus 100 nl of a reservoir containing 30% w/v polyethylene glycol 6000 and 0.1 M citrate buffer pH 5.0. PUUV Gn crystals were cryo-protected by immersion into a solution containing the crystallization reservoir plus 25% ethylene glycol and then rapidly cryo-cooled by immersion into liquid nitrogen.

Native data were collected at Diamond Light Source (DLS) beamline I03 to a resolution of 2.3 Å (λ = 0.9763). For phase determination, a crystal was soaked for 2 h with ~10 mM potassium tetrachloroplatinate (II), prior to cryo-protection. Diffraction data of the Pt-soaked crystal were collected at DLS beamline I04 at the L3 edge for platinum (λ = 1.0721) to a resolution of 3.7 Å. Diffraction images were indexed, integrated, and scaled with XIA2 (Winter, 2010). Five percent of reflections were randomly set aside to calculate the R_{free}. The single wavelength anomalous diffraction (SAD) method was employed for phase determination and heavy atom positions were identified, refined, and density modified in autoSHARP (de La Fortelle and Bricogne, 1997). The resulting low-resolution (3.7 Å) electron density map was used for initial building of the resulting two molecules in the asymmetric unit and a nearly complete model was built automatically using the high-resolution native data in autoBUILD (Terwilliger et al., 2008).

The structure of the Gn was refined using Refmac5 in the CCP4 suite (1994; Winn et al., 2003) and included iterative restrained refinement with automatically generated local non-crystallographic symmetry restraints and TLS (translation-libration-screw rotation) parameterization. Coot (Emsley and Cowtan, 2004) was used for manual rebuilding and MolProbity (Davis et al., 2007) was used to validate the model.

Sub-tomogram averaging

To locate spikes on the virions, the membrane of the virions was first modeled using either a sphere or an ellipsoid in Dynamo (Castano-Diez et al., 2012) *tomoview*, where evenly distributed and oriented pseudo-particles (denoted here as 'seeds') were created (Huiskonen et al., 2014). Seeds were defined with a seed-to-seed spacing of 28 pixels, resulting in an average of 600 seeds for each virus. 26,391 subtomograms were extracted from 44 virions at the seed locations into 160×160×160 voxels boxes. In the first stage of the iterative refinement scheme (Huiskonen et al., 2014), extracted densities were cylindrically averaged for each virus. The best average was used as an initial template to align all the seeds relative to the virion membrane allowing shifts only orthogonal to the plane of the membrane. At the end of each iteration, a custom post-processing Dynamo function was used to adjust the shift of each seed further by a weighting scheme taking the shifts of its neighbors into consideration as well. The contribution of each neighbor was weighted by a Gaussian function taking into account its distance to the seed in question, similarly to motion correction in single particle processing (Scheres, 2014). In the second stage, a previously determined TULV spike structure (EMDB-1704) (Huiskonen et al., 2010) was used as a template to locate the spikes on the virions. A shell-like mask encompassing a spike and some contribution from the adjacent four neighbors and two-fold symmetry was applied. The resolution was restricted to 43 Å for the first iteration and shifts of 28 pixels were allowed lateral to the membrane. At the end

of each iteration, a custom post-processing function was used to remove all seeds that were too close to each other except for the one with the best cross-correlation. In the third stage, all six parameters (three location coordinates and three Euler angles) were allowed to change simultaneously. The coordinates and orientations of particles were plotted and 1% of the particles whose coordinates and orientations deviated from the geometry of the virus were removed. Also, 10% of the particles with the bottom cross-correlation scores were removed. The resolution of the results was estimated by Fourier shell correlation (FSC) using a criterion of 0.143 (**Figure S4**).

To prevent over-refinement (correlation of noise instead of signal) during the refinement strategy described above, a custom post-processing function was designed to carry out 'gold-standard' refinements since the second stage, where the data is split into two independent sets and refinements were carried out independently. At the end of each iteration, the FSC of the two averages was calculated using a criterion of 0.143. After subtracting one shell from the corresponding value, the estimated resolution was used as low-pass filter for the next iteration.

Fitting PUUV Gn structure into the TULV reconstruction

For fitting of PUUV Gn, the TULV EM density map was segmented with Segger (Pintilie et al., 2010) in Chimera (Pettersen et al., 2004). The surface threshold was determined by matching the volume of one $(Gn–Gc)_4$ ectodomain spike to the theoretical volume corresponding to the calculated mass of the $(Gn–Gc)_4$ (416 kDa; constant protein density 0.81 Da/Å^3). The PUUV Gn crystal structure was converted to density by low pass filtering it to 16-Å resolution and 1,000 evenly rotated fits were considered for each segment (**Figure S4**). All fits in the central stalk region overlapped with their symmetry related copies (clash score >0.5) and were discarded from further analysis. Fitting to the membrane-distal lobes of the spike resulted in higher cross-correlation values than fitting to the other parts of the spike complex (**Figure S4**). From the pool of 1,000 evenly rotated fits, the best scoring unique fits were generated by using the 'optimize fits' option of Segger. Redundant fits less than 5 Å or 3 degrees apart were discarded.

SUPPLEMENTARY REFERENCES

(1994). The CCP4 suite: programs for protein crystallography. Acta Crystallogr D Biol Crystallogr *50*, 760-763.

Castano-Diez, D., Kudryashev, M., Arheit, M., and Stahlberg, H. (2012). Dynamo: a flexible, user-friendly development tool for subtomogram averaging of cryo-EM data in high-performance computing environments. J Struct Biol *178*, 139-151.

Chenna, R., Sugawara, H., Koike, T., Lopez, R., Gibson, T.J., Higgins, D.G., and Thompson, J.D. (2003). Multiple sequence alignment with the Clustal series of programs. Nucleic Acids Res *31*, 3497-3500.

Crispin, M., Bowden, T.A., Coles, C.H., Harlos, K., Aricescu, A.R., Harvey, D.J., Stuart, D.I., and Jones, E.Y. (2009). Carbohydrate and domain architecture of an immature antibody glycoform exhibiting enhanced effector functions. J Mol Biol *387*, 1061-1066.

Davis, I.W., Leaver-Fay, A., Chen, V.B., Block, J.N., Kapral, G.J., Wang, X., Murray, L.W., Arendall, W.B., 3rd, Snoeyink, J., Richardson, J.S*., et al.* (2007). MolProbity: all-atom contacts and structure validation for proteins and nucleic acids. Nucleic Acids Res *35*, W375-383.

de La Fortelle, E., and Bricogne, G. (1997). Maximum-likelihood heavy-atom parameter refinement for multiple isomorphous replacement and multiwavelength anomalous diffraction methods. In Methods in Enzymology, C.C.a.S. RM, ed. (New York: Academic Press), pp. 472-494.

Edgar, R.C. (2004). MUSCLE: multiple sequence alignment with high accuracy and high throughput. Nucleic Acids Res *32*, 1792-1797.

Emsley, P., and Cowtan, K. (2004). Coot: model-building tools for molecular graphics. Acta Crystallogr D Biol Crystallogr *60*, 2126-2132.

Gouet, P., Courcelle, E., Stuart, D.I., and Metoz, F. (1999). ESPript: analysis of multiple sequence alignments in PostScript. Bioinformatics *15*, 305-308.

Heiskanen, T., Lundkvist, A., Soliymani, R., Koivunen, E., Vaheri, A., and Lankinen, H. (1999). Phage-displayed peptides mimicking the discontinuous neutralization sites of puumala Hantavirus envelope glycoproteins. Virology *262*, 321-332.

Hepojoki, J., Strandin, T., Vaheri, A., and Lankinen, H. (2010). Interactions and oligomerization of hantavirus glycoproteins. Journal of virology *84*, 227-242.

Horling, J., and Lundkvist, A. (1997). Single amino acid substitutions in Puumala virus envelope glycoproteins G1 and G2 eliminate important neutralization epitopes. Virus Res *48*, 89-100.

Huiskonen, J.T., Hepojoki, J., Laurinmaki, P., Vaheri, A., Lankinen, H., Butcher, S.J., and Grunewald, K. (2010). Electron cryotomography of Tula hantavirus suggests a unique assembly paradigm for enveloped viruses. J Virol *84*, 4889-4897.

Huiskonen, J.T., Parsy, M.L., Li, S., Bitto, D., Renner, M., and Bowden, T.A. (2014). Averaging of viral envelope glycoprotein spikes from electron cryotomography reconstructions using Jsubtomo. J Vis Exp, e51714.

Jenison, S., Yamada, T., Morris, C., Anderson, B., Torrez-Martinez, N., Keller, N., and Hjelle, B. (1994). Characterization of human antibody responses to four corners hantavirus infections among patients with hantavirus pulmonary syndrome. Journal of virology *68*, 3000-3006.

Laskowski, R.A. (2009). PDBsum new things. Nucleic Acids Res *37*, D355-359.

Pettersen, E.F., Goddard, T.D., Huang, C.C., Couch, G.S., Greenblatt, D.M., Meng, E.C., and Ferrin, T.E. (2004). UCSF Chimera--a visualization system for exploratory research and analysis. J Comput Chem *25*, 1605-1612.

Pintilie, G.D., Zhang, J., Goddard, T.D., Chiu, W., and Gossard, D.C. (2010). Quantitative analysis of cryo-EM density map segmentation by watershed and scale-space filtering, and fitting of structures by alignment to regions. J Struct Biol *170*, 427-438.

Scheres, S.H. (2014). Beam-induced motion correction for sub-megadalton cryo-EM particles. Elife *3*, e03665.

Shi, X., and Elliott, R.M. (2004). Analysis of N-linked glycosylation of hantaan virus glycoproteins and the role of oligosaccharide side chains in protein folding and intracellular trafficking. J Virol *78*, 5414-5422.

Terwilliger, T.C., Grosse-Kunstleve, R.W., Afonine, P.V., Moriarty, N.W., Zwart, P.H., Hung, L.W., Read, R.J., and Adams, P.D. (2008). Iterative model building, structure refinement and density modification with the PHENIX AutoBuild wizard. Acta Crystallogr D Biol Crystallogr *64*, 61-69.

Walter, T.S., Diprose, J.M., Mayo, C.J., Siebold, C., Pickford, M.G., Carter, L., Sutton, G.C., Berrow, N.S., Brown, J., Berry, I.M.*, et al.* (2005). A procedure for setting up high-throughput nanolitre crystallization experiments. Crystallization workflow for initial screening, automated storage, imaging and optimization. Acta Cryst D *61*, 651-657.

Winn, M.D., Murshudov, G.N., and Papiz, M.Z. (2003). Macromolecular TLS refinement in REFMAC at moderate resolutions. Methods Enzymol *374*, 300-321.

Winter, G. (2010). xia2: an expert system for macromolecular crystallography data reduction. Journal of Applied Crystallography *43*, 186-190.
